# Supplementary material for: A Systematic Study on the Degradation Products Generated from Artificially Aged Microplastics
Source: Polymers (Basel). 2021 Jun 18;13(12):1997. doi: 10.3390/polym13121997 (PMC8234390; doi:10.3390/polym13121997)
Supplement: Supplementary file 1 [file polymers-13-01997-s001.zip › polymers-1214266-supplementary.pdf]

A systematic study on the degradation products generated from artificially aged microplastics

Greta Biale[1], Jacopo La Nasa[1,2*], Marco Mattonai[1], Andrea Corti[1], Virginia Vinciguerra[1], Valter Castelvetro[1,3], Francesca Modugno[1,3]

[1]*Department of Chemistry and Industrial Chemistry, University of Pisa, Pisa, Italy*
[2]*National Interuniversity Consortium of Materials Science and Technology, Florence, Italy*
[3]*CISUP—Center for the Integration of Scientific Instruments of the University of Pisa, University of Pisa, Pisa, Italy*

Supplementary Materials

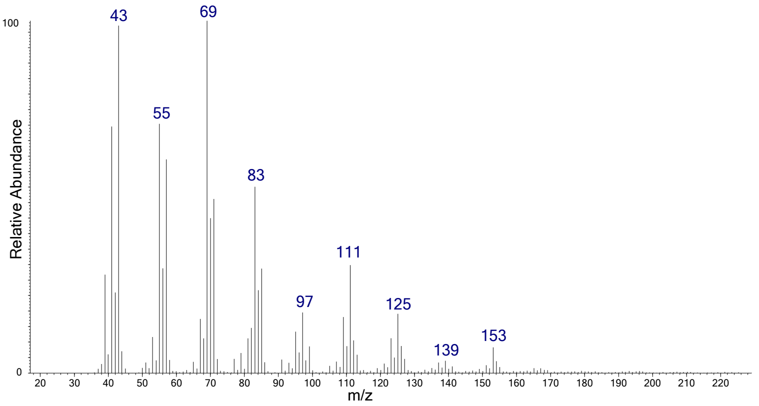

Figure S.1 Average mass spectrum of the peak (from 421 °C to 480 °C) from the EGA curve (**Figure 3**) of the unaged polypropylene (PP-0w) reference sample.

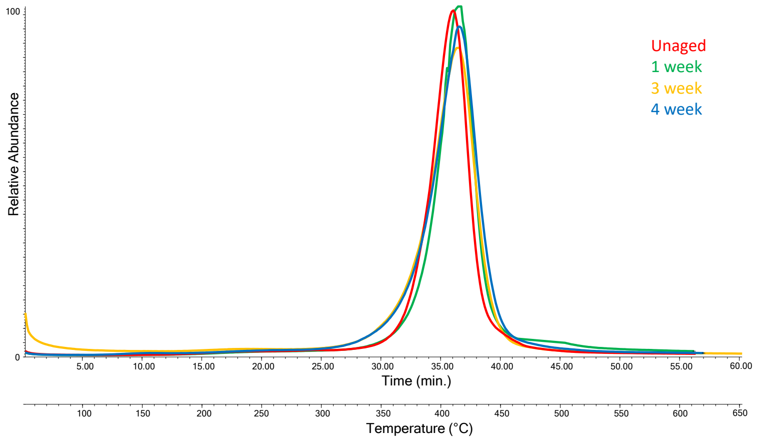

Figure S.2 EGA profiles of PS after different artificial aging times: PS-0w (red), PS-1w (green), PS-3w (yellow), and PS-4w (blue) accelerated aging.

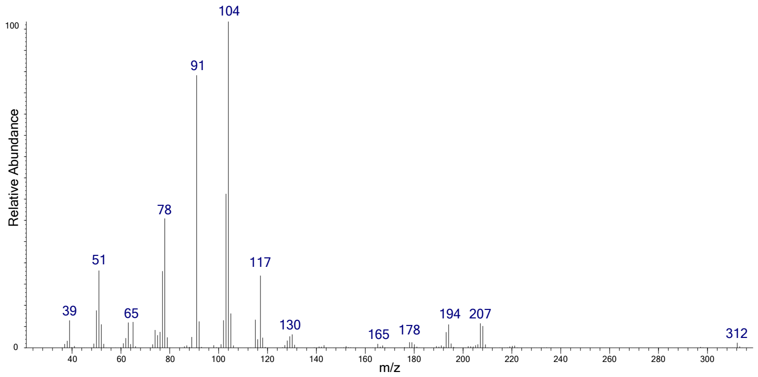

Figure S.3 Average mass spectrum of the peak (from 350 °C to 450 °C) from the EGA curve (**Figure S.2**) of the unaged polystyrene (PS-0w) reference sample.

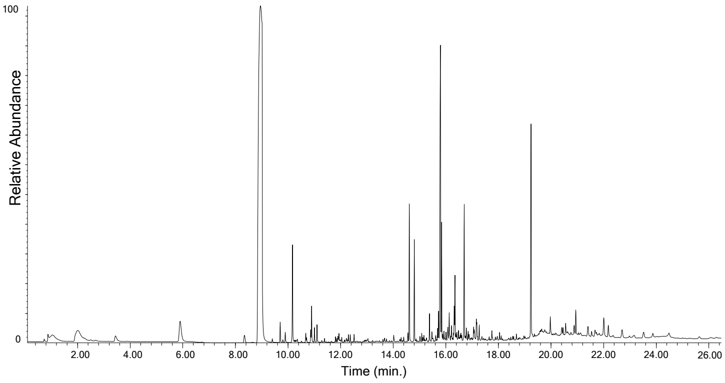

Figure S.4 Chromatogram obtained in the Py-GC-MS analysis of PS-4w.

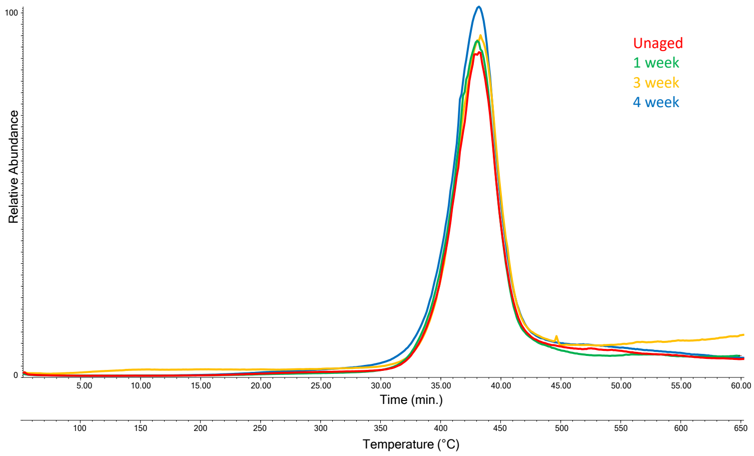

Figure S.5 EGA profiles of PET after different artificial aging times: PET-0w (red), PET-1w (green), PET-3w (yellow), and PET-4w (blue) accelerated aging.

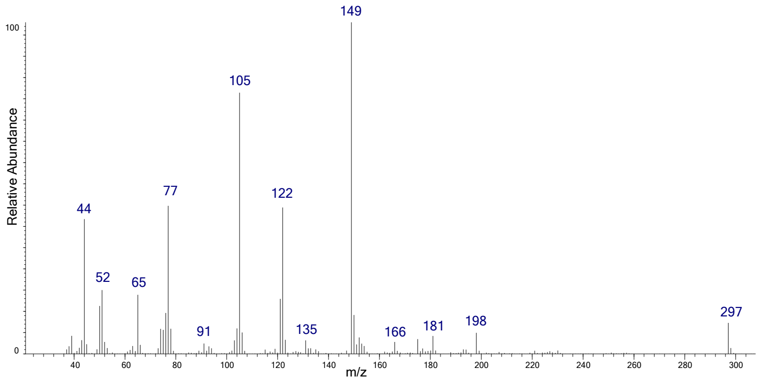

Figure S.6 Average mass spectrum of the peak (from 381 °C to 463 °C) from the EGA curve (**Figure S.5**) of the unaged polyethylene terephthalate (PET-0w) reference sample.

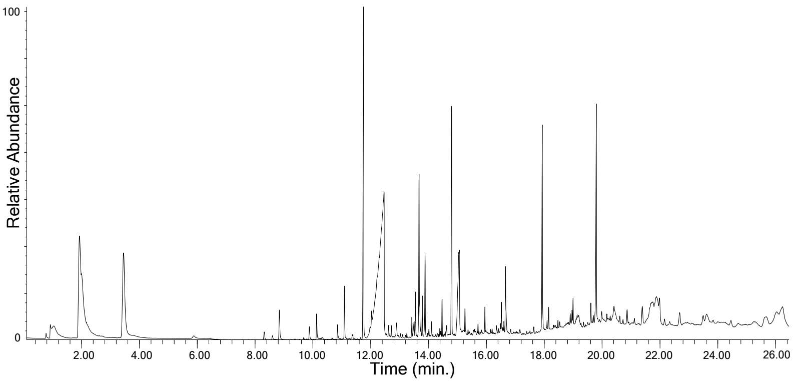

Figure S.7 Chromatogram obtained in the Py-GC-MS analysis of PET-4w.

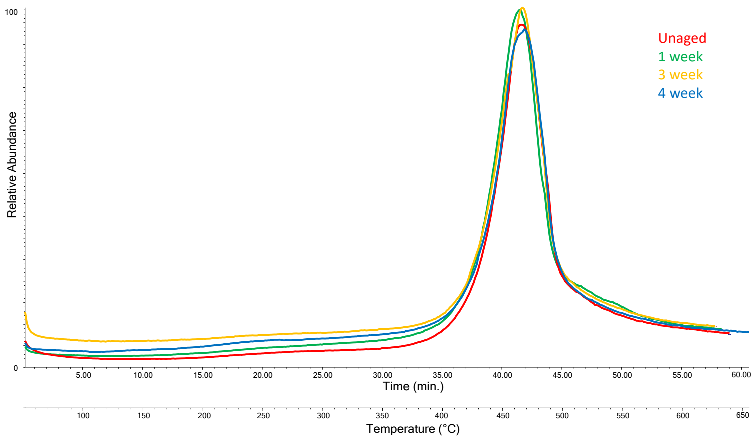

Figure S.8 EGA profiles of LDPE after different artificial aging times: LDPE-0w (red), LDPE-1w (green), LDPE-3w (yellow), and LDPE-4w (blue) accelerated aging.

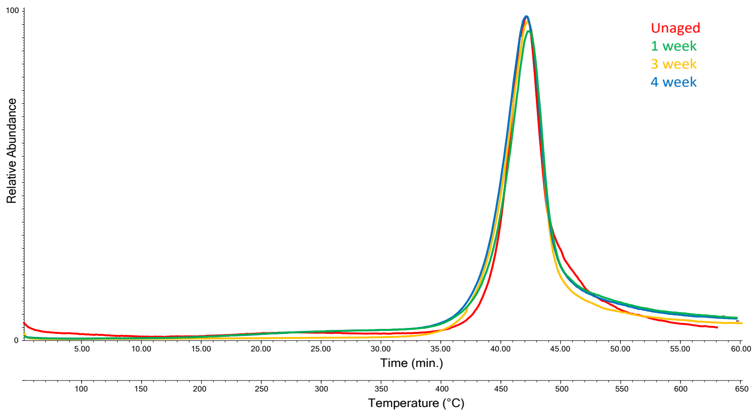

Figure S.9 EGA profiles of HDPE after different artificial aging times: HDPE-0w (red), HDPE-1w (green), HDPE-3w (yellow), and HDPE-4w (blue) accelerated aging.

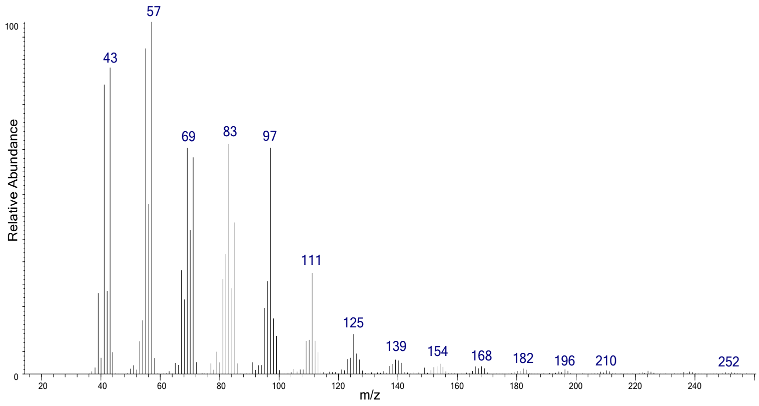

Figure S.10 Average mass spectrum of the peak (from 424 °C to 499 °C) from the EGA curve (**Figure S.8**) of the unaged low-density polyethylene (LDPE-0w) reference sample.

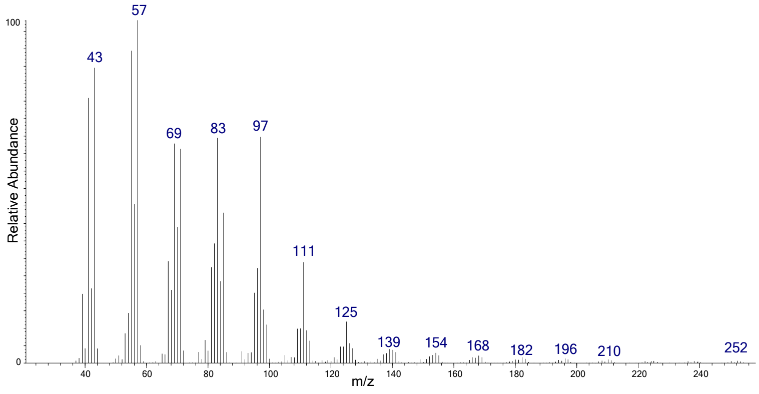

Figure S.11 Average mass spectrum of the peak (from 439 °C to 504 °C) from the EGA curve (**Figure S.9**) of the unaged high-density polyethylene (HDPE-0w) reference sample.

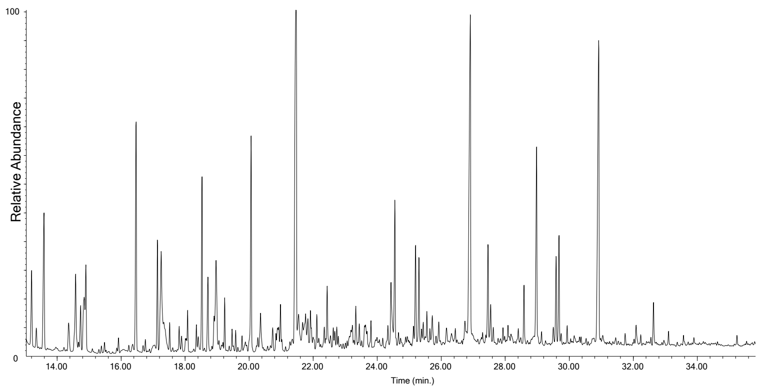

Figure S.12 Chromatogram obtained in the Py(HMDS)-GC-MS analysis of the DCM extract of PP-0w. The list of the main pyrolysis products is reported in **Table S.8**.

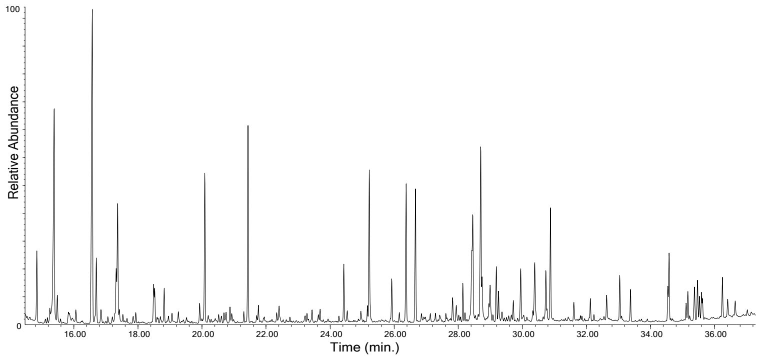

Figure S.13 Chromatogram obtained in the Py(HMDS)-GC-MS analysis of the MeOH extract of PS-0w. List of the main pyrolysis products is reported in **Table S.9**.

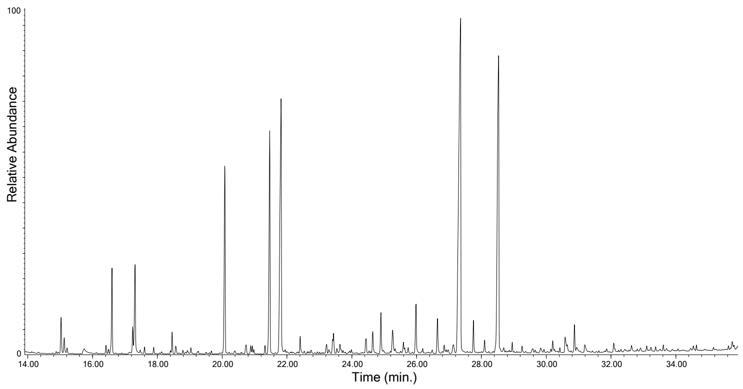

Figure S.14 Chromatogram obtained in the Py(HMDS)-GC-MS analysis of the DCM extract of PET-0w.

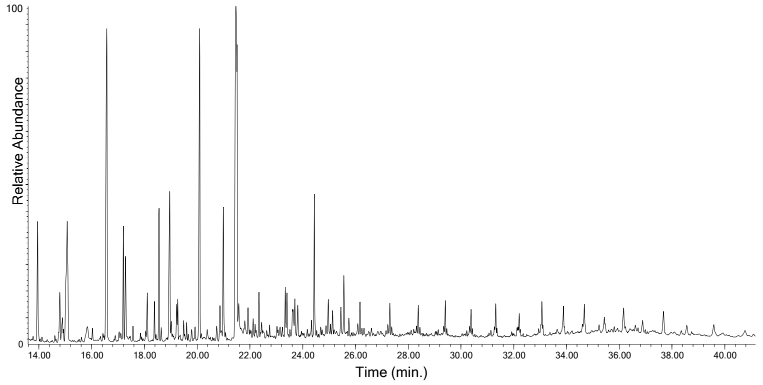

Figure S.15 Chromatogram obtained in the Py(HMDS)-GC-MS analysis of the DCM extract of LDPE-0w. The list of the main pyrolysis products is reported in **Table S.11**.

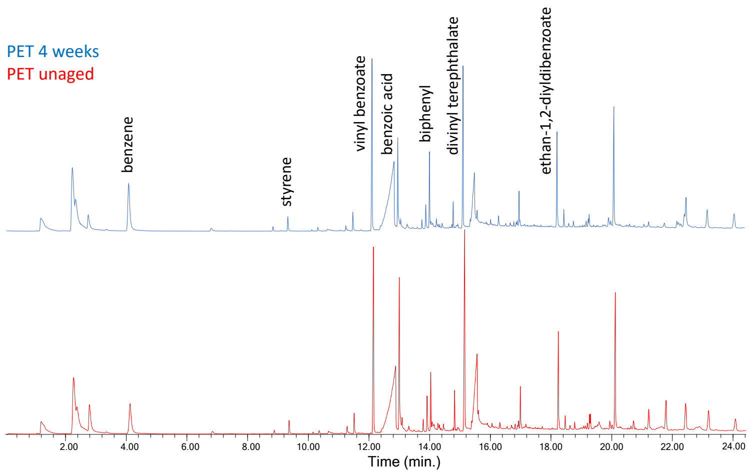

Figure S.16 Chromatograms obtained in the Py-GC-MS analysis of the extraction residues of the PET-0w (red) and PET-4w (blue). The complete list of the main pyrolysis products is reported in **Table S.16** in the Appendix.

Table S.1 Identification of peaks in the chromatogram obtained in the Py-GC-MS analysis of PP-0w (Figure 4). M+ refers to the molecular ion.

#	t_r (min)	Peak identification	Main ions (*m*/*z*)
1	1.9	propene	42 (M+), **41**, 39
2	2.2	pentane	72 (M+), 57, **43**
3	2.7	2,methyl-1-pentene	84 (M+), 69, **56**, 41
4	5.6	4-methyl-2-heptene	112 (M+), **69**, 55, 41
5	6.2	2-methyl-1,5-hexadiene	**95**, 81, 67, 55, 39
6	8.0	2,4-dimethyl-1-heptene	126 (M+), 83, 70, 55, **43**
7	8.2	1,3,5-trimethylcyclohexane (isomer)	126, **111**, 69, 55, 41
8	8.5	2,4-dimethyl-1,6-heptadiene	124 (M+), **109**, 81, 67, 55, 41
9	8.9	2,4,6-trimethyl-1-heptene	140 (M+), 83, 69, 55, **43**
10	9.2	2,4,6-trimethyl-1,6-heptadiene	138 (M+), 123, 109, 95, **82**, 67, 55, 41
11	10.3	4,6-dimethyl-2-nonene	154 (M+), 111, 85, **69**, 55, 41
12	11.1	2,4,6-trimethyl-1-nonene	168 (M+), 125, 111, 83, **69**, 57, 43

13	11.2	2,4,6-trimethyl-1-nonene	168 (M+), 125, 111, 83, **69**, 57, 43
14	11.6	2,4,6,8-tetramethyl-1-nonene	182 (M+), 125, 111, 83, **69**, 57, 43
15	11.8	2,4,6,8-tetramethyl-1,8-nonadiene	180 (M+), 123, 109, 96, 83, **69**, 55, 41
16	13.0	2,4,6,8-tetramethyl-1-undecene	210 (M+), 154, 111, 83, **69**, 55, 43
17	13.1	2,4,6,8-tetramethyl-1-undecene	210 (M+), 154, 111, 85, **69**, 55, 43
18	13.2	2,4,6,8-tetramethyl-1-undecene	210 (M+), 154, 111, 85, **69**, 55, 44
19	13.3	2,4,6,8,10-pentamethyl-1-undecene	224 (M+), 125, 111, 97, 83, **69**, 57, 43
20	13.6	2,4,6,8,10-pentamethyl-1,10-undecadiene	222 (M+), 123, 109, 95, 83, **69**, 55, 41
21	13.9	unknown	153, 125, 111, 97, 85, **69**, 57, 43
22	14.3	2,4,6,8,10-pentamethyl-1,12-tridecadiene	250 (M+), 123, 109, 95, 83, **69**, 55, 41
23	14.5	2,4,6,8,10-pentamethyl-1-tridecene	252 (M+), 125, 111, 97, 83, **69**, 57, 43
24	14.6	unknown	196, 125, 111, 97, 83, **69**, 57, 43
25	14.7	unknown	153, 125, 111, 97, 83, **69**, 57, 43
26	14.9	2,4,6,8,10,12-hexamethyl-1,12-tridecadiene	264 (M+), 123, 109, 95, 83, **69**, 55, 41
27	15.6	2,4,6,8,10,12-hexamethyl-1,14-pentadecadiene	292 (M+), 137, 109, 97, 83, **69**, 55, 41
28	15.7	2,4,6,8,10,12-hexamethyl-1-pentadecene	294 (M+), 139, 111, 97, 83, **69**, 57, 43
29	15.9	unknown	294, 139, 125, 111, 97, 83, **69**, 57, 43
30	16.1	2,4,6,8,10,12,14-heptamethyl-1,14-pentadecadiene	306 (M+), 123, 109, 97, 83, **69**, 55, 41
31	16.8	2,4,6,8,10,12,14-heptamethyl-1,16-heptadecadiene	334 (M+), 123, 109, 97, 83, **69**, 55, 41
32	16.9	2,4,6,8,10,12,14-heptamethyl-1-heptadecene	336 (M+), 125, 111, 97, 83, **69**, 57, 43
33	17.0	unknown	153, 125, 111, 97, 83, **69**, 57, 43
34	17.1	unknown	153, 125, 111, 97, 83, **69**, 57, 43
35	17.2	2,4,6,8,10,12,14,16-octamethyl-1,16-heptadecadiene	348 (M+), 123, 109, 97, 83, **69**, 55, 41
36	17.8	2,4,6,8,10,12,14,16-octamethyl-1,18-nonadecadiene	376 (M+), 125, 109, 97, 83, **69**, 55, 41
37	17.9	2,4,6,8,10,12,14,16-octamethyl-1-nonadecene	378 (M+), 125, 111, 97, 83, **69**, 57, 43
38	18.2	2,4,6,8,10,12,14,16,18-nonamethyl-1,18-nonadecadiene	390 (M+) 125, 109, 97, 83, **69**, 55, 41
39	18.7	2,4,6,8,10,12,14,16,18-nonamethyl-1,20-henicosadiene	418 (M+), 125, 111, 97, 83, **69**, 55, 41
40	18.8	2,4,6,8,10,12,14,16,18-nonamethyl-1-henicosene	420 (M+), 125, 111, 97, 83, **69**, 57, 43
41	19.1	2,4,6,8,10,12,14,16,18,20-decamethyl-1,20-henicosadiene	432 (M+), 125, 109, 97, 83, **69**, 55, 43
42	19.6	2,4,6,8,10,12,14,16,18,20-decamethyl-1,22-tricosadiene	460 (M+), 125, 111, 97,83, **69**, 55, 41
43	19.7	2,4,6,8,10,12,14,16,18,20-decamethyl-1-tricosene	153, 139, 125, 111, 97, 83, **69**, 57, 43
44	20.0	2,4,6,8,10,12,14,16,18,20,22-undecamethyl-1,22-tricosadiene	474 (M+), 125, 111, 97, 83, **69**, 55, 43

Table S.2 Identification of peaks in the chromatogram obtained in the Py-GC-MS analysis of PS-0w (Figure 5).

#	t_r (min)	Peak identification	Main ions (*m*/*z*)
1	6.0	toluene	92, **91**, 65
2	8.9	styrene	**104**, 78, 63, 51
3	9.7	allylbenzene	**117**, 103, 91, 65
4	9.9	benzaldehyde	106, **105**, 77, 51
5	10.2	α-methylstyrene	**118**, 103, 91, 78, 63, 51
6	10.8	3-butenylbenzene	132, 104, **91**, 65
7	11.0	(1-methylenepropyl)-benzene	132, **117**, 103, 91, 77, 63, 51
8	11.1	acetophenone	120, **105**, 91, 77, 51

9	14.6	1,2-diphenylethane	182, **91**, 65
10	14.8	propane-1,2-diyldibenzene	196, **105**, 91, 77, 65
11	15.4	1,1'-(1,3-propanediyl)bis-benzene	196, 105, **92**, 77, 65, 51
12	15.7	stilbene	180, **179**, 165, 152, 102, 89, 76
13	15.8	3-butene-1,3-diyldibenzene (styrene dimer)	208, 193, 130, 115, 104, **91**, 77, 65
14	15.9	1-pentene-2,4-diyldibenzen	222, 194, 179, 115, **105**, 91, 77
15	16.3	(E)-1-butene-1,4-diyldibenzene	208, **117**, 115, 91, 65
16	16.7	hexa-1,5-diene-2,5-diyldibenzene	234, 143, **130**, 115, 104, 91, 77, 65
17	19.2	5-hexene-1,3,5-triyltribenzene (styrene trimer)	312, 207, 194, 117, **91**, 77

Table S.3 Identification of peaks in the chromatogram obtained in the Py-GC-MS analysis of PET-0w (Figure 6).

#	t_r (min)	Peak identification	Main ions (*m/z*)
1	2.0	carbon dioxide	**44**
2	3.5	benzene	**78**, 63, 51
3	8.3	ethylbenzene	106, **91**, 77, 65, 51
4	8.9	styrene	**104**, 78, 63, 51
5	9.9	benzaldehyde	106, **105**, 77, 51
6	10.1	phenol	**94**, 79, 66, 55, 39
7	10.9	benzeneacetaldehyde	120, **91**, 65, 51
8	11.1	acetophenone	120, **105**, 77, 51
9	11.8	vinyl benzoate	148, **105**, 77, 51
10	12.4	benzoic acid	122, **105**, 77, 51
11	13.7	biphenyl	**154**, 131, 115, 76
12	14.8	divinyl terephthalate	**175**, 147, 132, 104, 76
13	15.1	4-(vinyloxycarbonyl) benzoic acid	**149**, 121, 76, 65
14	15.2	benzophenone	182, 152, **105**, 77, 51
15	15.7	stilbene	**180**, 179, 165, 152, 102, 89, 76
16	15.9	9H-fluoren-9-one	**180**, 152, 126, 76
17	16.7	unknown	198, **181**, 152, 76
18	17.9	ethan-1,2-divinyldibenzoate	227, **105**, 77, 51
19	18.2	unknown	**230**, 198, 181, 152, 115
20	19.0	unknown	296, 149, **131**, 105, 77, 51
21	19.8	2-(benzoyloxy) ethyl vinyl terephthalate	**297**, 149, 105, 77
22	22.0	ethan-1,2-diyl divinyl diterephthalate	**367**, 325, 296, 175, 162, 104

Table S.4 Identification of peaks in the chromatogram obtained in the Py-GC-MS analysis of LDPE-0w (Figure 7). M+ refers to the molecular ion.

#	tr (min)	Peak identification	Main ions (m/z)
1	2.7	1-hexene	84 (M+), 69, **56**, 41
2	3.9	1-heptene	98 (M+), 83, 70, **56**, 41
3	4.0	heptane	100 (M+), 71, 57, **43**
4	6.7	1-octene	112 (M+), 97, 83, 70, **55**, 41
5	6.9	octane	114 (M+), 85, 71, 57, **43**
6	8.7	1,8-nonadiene	109, 96, 81, **67**, 55, 41
7	8.8	1-nonene	126 (M+), 97, 83, 69, **56**, 41
8	9.0	nonane	128 (M+), 99, 85, 71, 57, **43**
9	10.1	1,9-decadiene	110, 95, 81, 67, **55**, 41

10	10.2	1-decene	140 (M+), 111, 97, 83, 70, 55, **41**
11	10.3	decane	142 (M+), 117, 99, 85, 71, **57**, 43
12	11.2	1,10-undecadiene	124, 109, 95, 81, 67, 55, **41**
13	11.3	1-undecene	154 (M+), 126, 111, 97, 83, 70, 55, **43**
14	11.4	undecane	156 (M+), 98, 85, 71, **57**, 43
15	12.1	1,11-dodecadiene	138, 124, 109, 95, 81, 67, **55**, 41
16	12.13	1-dodecene	168 (M+), 140, 125, 111, 97, 83, 69, 55, **41**
17	12.2	dodecane	170 (M+), 128, 85, 71, **57**, 43
18	12.3	decanal	138, 128, 112, 95, 82, 68, 55, **41**
19	12.8	1,12-tridecadiene	123, 109, 95, 81, 67, **55**, 41
20	12.9	1-tridecene	125, 111, 97, 83, 69, 55, **41**
21	13.0	tridecane	184 (M+), 99, 85, 71, **57**, 43
22	13.05	undecanal	152, 126, 109, 96, 82, 68, 55, **41**
23	13.5	1,13-tetradecadiene	123, 109, 96, 81, 67, **55**, 41
24	13.6	1-tetradecene	125, 111, 97, 83, 69, **55**, 41
25	13.64	tetradecane	198 (M+), 99, 85, 71, **57**, 43
26	13.72	dodecanal	166, 140, 123, 110, 96, 82, 68, **57**, 41
27	14.2	1,14-pentadecadiene	123, 109, 96, 81, 67, **55**, 41
28	14.25	1-pentadecene	210 (M+), 125, 111, 97, 83, 69, **55**, 41
29	14.3	pentadecane	212 (M+), 113, 99, 85, 71, **57**, 43
30	14.4	tridecanal	180, 154, 124, 110, 96, 82, 68, **57**, 43
31	14.8	1,15-hexadecadiene	123, 109, 96, 82, 67, **55**, 41
32	14.86	1-hexadecene	224 (M+), 125, 111, 97, 83, 69, **55**, 41
33	14.9	hexadecane	226 (M+), 99, 85, 71, **57**, 43
34	15.0	tetradecanal	194, 168, 138, 110, 96, 82, 69, **57**, 41
35	15.4	1,16-heptadecadiene	137, 123, 109, 96, 82, 69, **55**, 41
36	15.44	1-heptadecene	238 (M+), 139, 125, 111, 97, 83, 69, **55**, 41
37	15.5	heptadecane	240 (M+), 99, 85, 71, **57**, 43
38	15.6	pentadecanal	208, 180, 111, 96, 82, 69, 57, **41**
39	15.9	1,17-octadecadiene	123, 109, 96, 82, 69, **55**, 41
40	16.0	1-octadecene	252 (M+), 125, 111, **97**, 83, 69, 55, 41
41	16.02	octadecane	254 (M+), 99, 85, 71, **57**, 43
42	16.1	hexadecanal	222, 124, 111, 96, 82, 69, **57**, 43
43	16.5	1,18-nonadecadiene	137, 123, 109, 96, 82, 69, **55**, 41
44	16.51	1-nonadecene	139, 125, 111, 97, **83**, 69, 55, 43
45	16.54	nonadecane	268 (M+), 127, 113, 99, 85, 71, **57**, 43
46	16.7	heptadecanal	236, 208, 137, 124, 109, 96, **82**, 68, 57, 41
47	16.97	1,19-eicosadiene	137, 123, 109, 96, 82, 69, **55**, 41
48	17.01	1-eicosene	139, 125, 111, **97**, 83, 69, 55, 43
49	17.04	eicosane	127, 113, 99, 85, 71, **57**, 43
50	17.1	octadecanal	250, 137, 124, 109, 96, **82**, 68, 57, 43
51	17.4	1,20-heneicosadiene	137, 123, 109, 96, 82, 69, **55**, 41
52	17.48	1-heneicosene	139, 125, 111, **97**, 83, 69, 55, 43
53	17.50	heneicosane	113, 97, 85, 71, **57**, 43
54	17.6	nonadecanal	264, 202, 137, 124, 110, 97, **82**, 69, 57, 43
55	17.91	1,21-docosadiene	151, 137, 123, 109, 96, 82, 67, **55**, 41
56	17.94	1-docosene	139, 125, 111, **97**, 83, 69, 55, 43
57	17.96	docosane	310 (M+), 99, 85, 71, **57**, 43
58	18.1	eicosanal	278, 250, 123, 111, 96, 82, 69, **57**, 43
59	18.3	1,22-tricosadiene	137, 123, 109, 96, 82, 69, **55**, 41
60	18.37	1-tricosene	322 (M+), 125, 111, **97**, 83, 69, 55, 43
61	18.39	tricosane	324 (M+), 113, 97, 85, 71, **57**, 43

62	18.5	heneicosanal	292, 138, 123, 111, 96, 82, 68, **57**, 43
63	18.77	1,23-tetracosadiene	334 (M+), 137, 123, 109, 96, 82, 69, **55**, 41
64	18.8	1-tetracosene	336 (M+), 139, 125, 111, **97**, 83, 69, 57, 43
65	19.0	docosanal	306, 251, 138, 124, 111, 96, 82, 71, **57**, 43
66	19.2	1-pentacosene	350 (M+), 139, 125, 111, **97**, 83, 69, 57, 43
67	19.35	tricosanal	320, 139, 125, 111, 97, 83, 71, **57**, 43
68	19.6	1-hexacosene	364 (M+), 139, 125, 111, **97**, 83, 69, 57, 43
69	19.8	unknown	**297**, 149, 105, 77

Table S.5 Identification of peaks in the chromatogram obtained in the Py-GC-MS analysis of LDPE-4w.

#	t_r	Peak identification	Main ions (*m*/*z*)
1	2.7	1-hexene	84, 69, **56**, 41
2	3.9	1-heptene	98, 83, 70, **56**, 41
3	4.0	heptane	100, 71, 57, **43**
4	6.7	1-octene	112, 97, 83, 70, **55**, 41
5	6.9	octane	114, 85, 71, 57, **43**
6	8.7	1,8-nonadiene	109, 96, 81, **67**, 55, 41
7	8.9	1-nonene	126, 97, 83, 69, **56**, 41
8	9.0	nonane	128, 99, 85, 71, 57, **43**
9	10.1	1,9-decadiene	110, 95, 81, 67, **55**, 41
10	10.2	1-decene	140, 111, 97, 83, 70, 55, **41**
11	10.35	decane	142, 113, 99, 85, 71, **57**, 43
12	11.2	1,10-undecadiene	124, 109, 95, 81, 67, 55, **41**
13	11.3	1-undecene	154, 126, 111, 97, 83, 70, 55, **41**
14	11.36	undecane	156, 98, 85, 71, **57**, 43
15	12.1	1,11-dodecadiene	138, 124, 109, 95, 81, 67, **55**, 41
16	12.15	1-dodecene	168, 140, 125, 111, 97, 83, 69, 55, **41**
17	12.2	dodecane	170, 127, 85, 71, **57**, 43
18	12.3	decanal	138, 128, 112, 95, 82, 70, 57, **41**
19	12.7	nonanoic acid	158, 129, 115, 98, 85, 73, **57**, 41
20	12.8	1,12-tridecadiene	123, 109, 95, 81, 67, **55**, 41
21	12.9	1-tridecene	125, 111, 97, 83, 69, **55**, 41
22	13.0	tridecane	184, 99, 85, 71, **57**, 43
23	13.03	undecanal	142, 126, 109, 96, 82, 68, 57, **41**
24	13.4	decanoic acid	172, 129, **73**, 55, 41
25	13.48	undecanol	126, 111, 97, **85**, 69, 55, 43
26	13.5	1,13-tetradecadiene	123, 109, 96, 81, 67, **55**, 41
27	13.6	1-tetradecene	125, 111, 97, 83, 69, **55**, 41
28	13.66	tetradecane	198, 99, 85, 71, **57**, 43
29	13.7	dodecanal	166, 140, 123, 110, 96, 82, 68, **57**, 41
30	14.05	undecanoic acid	186, 143, 129, 115, 83, **73**, 60, 41
31	14.1	dodecanol	141, 127, 111, 97, 83, 69, **57**, 41
32	14.2	1,14-pentadecadiene	123, 109, 96, 82, 67, **55**, 41
33	14.26	1-pentadecene	210, 125, 111, 97, 83, 69, **55**, 41
34	14.3	pentadecane	141, 127, 113, 99, 85, 71, **57**, 43
35	14.4	tridecanal	180, 154, 124, 110, 96, 82, 68, **57**, 41
36	14.6	dodecanoic acid	200, 157, 129, 115, 98, 85, **73**, 60, 43
37	14.77	tridecanol	182, 155, 141, 125, 111, 97, 83, 69, 55, **43**
38	14.8	1,15-hexadecadiene	123, 109, 96, 82, 69, **55**, 41
39	14.87	1-hexadecene	224, 125, 111, 97, 83, 69, **55**, 41

40	14.9	hexadecane	226, 99, 85, 71, **57**, 43
41	15.0	tetradecanal	194, 168, 138, 124, 109, 96, 82, 69, **57**, 41
42	15.2	tridecanoic acid	214, 185, 171, 157, 143, 129, 115, 85, **73**, 60
43	15.37	tetradecanol	169, 125, 111, 97, 83, 69, **55**, 41
44	15.4	1,16-heptadecadiene	137, 123, 109, 96, 82, 69, **55**, 41
45	15.45	1-heptadecene	238, 139, 125, 111, 97, **83**, 69, 55, 41
46	15.5	heptadecane	240, 99, 85, 71, **57**, 43
47	15.6	pentadecanal	208, 182, 109, 96, **82**, 69, 57, 43
48	15.8	tetradecanoic acid	228, 185, 129, **73**
49	15.93	pentadecanol	183, 125, 111, 97, 83, 70, **57**, 41
50	15.96	1,17-octadecadiene	123, 109, 96, 82, 69, **55**, 41
51	16.0	1-octadecene	252, 125, 111, 97, **83**, 69, 55, 43
52	16.03	octadecane	254, 99, 85, 71, **57**, 43
53	16.05	2-hexadecanone	240, 111, 97, 85, 71, 58, **43**
54	16.1	hexadecanal	222, 123, 109, 96, **82**, 68, 57, 43
55	16.3	pentadecanoic acid	242, 199, 129, **73**, 57
56	16.46	hexadecanol	197, 125, 111, 97, 83, 69, **55**, 41
57	16.5	1,18-nonadecadiene	137, 123, 109, 96, 82, 69, **55**, 41
58	16.53	1-nonadecene	139, 125, 111, **97**, 83, 69, 55, 43
59	16.55	nonadecane	268, 127, 113, 99, 85, 71, **57**, 43
60	16.58	2-heptadecanone	254, 85, 71, **58**, 43
61	16.7	heptadecanal	236, 208, 137, 123, 109, 96, **82**, 68, 57, 43
62	16.8	hexadecanoic acid	256, 213, 129, **73**, 55, **43**
63	16.99	1,19-eicosadiene	137, 123, 109, 96, 82, 69, **55**, 41
64	17.0	1-eicosene	139, 125, 111, **97**, 83, 69, 55, 43
65	17.04	eicosane	127, 113, 99, 85, 71, **57**, 43
66	17.09	2-octadecanone	268, 114, 96, 85, 71, **58**, 43
67	17.2	octadecanal	250, 137, 124, 109, 96, **82**, 68, 57, 43
68	17.3	heptadecanoic acid	270, 227, 185, 171, 129, **70**, **55**
69	17.47	1,20-heneicosadiene	137, 123, 109, 96, 82, 69, **55**, 41
70	17.5	1-heneicosene	139, 125, 111, **97**, 83, 69, 55, 43
71	17.52	heneicosane	113, 99, 85, 71, **57**, 43
72	17.56	2-nonadecanone	282, 127, 113, 95, 85, 71, **58**, 43
73	17.65	nonadecanal	264, 236, 137, 123, 109, 96, **82**, 69, 57, 43
74	17.8	octadecanoic acid	284, 241, 185, 129, 97, **57**
75	17.92	1,21-docosadiene	137, 123, 109, 96, 82, 69, **55**, 41
76	17.95	1-docosene	139, 125, 111, **97**, 83, 69, 57, 43
77	17.97	docosane	310, 99, 85, 71, **57**, 43
78	18.02	2-eicosanone	296, 127, 96, 85, 71, **58**, 43
79	18.1	eicosanal	278, 250, 123, 109, 96, **82**, 69, 57, 43
80	18.2	nonadecanoic acid	298, 255, 129, 83, 69, **57**, 43
81	18.36	1,22-tricosadiene	137, 123, 109, 96, 82, 69, **55**, 41
82	18.39	1-tricosene	322, 125, 111, **97**, 83, 69, 57, 43
83	18.40	tricosane	324, 113, 97, 85, 71, **57**, 43
84	18.46	2-heneicosanone	310, 127, 109, 96, 82, 71, **59**, 43
85	18.5	heneicosanal	292, 138, 123, 111, 96, **82**, 69, 57, 43
86	18.78	1,23-tetracosadiene	334, 137, 123, 109, 96, 82, 69, **55**, 41
87	18.8	1-tetracosene	336, 139, 125, 111, **97**, 83, 69, 57, 43
88	18.9	2-docosanone	324, 309, 109, 96, 82, 71, **58**, 43
89	19.0	docosanal	306, 250, 138, 123, 109, 96, **82**, 69, 57, 43
90	19.2	1-pentacosene	350, 139, 125, 111, **97**, 83, 69, 57, 43
91	19.4	tricosanal	320, 137, 124, 109, 96, **82**, 68, 57, 43

92	19.6	1-hexacosene	364, 139, 125, 111, 97, 83, 69, **57**, 43
93	19.8	tetracosanal	334, 138, 123, 109, 96, **82**, 69, 57, 43
94	20.0	heptacosane	380, 125, 111, 97, 85, 71, **57**, 43
95	20.2	pentacosanal	348, 125, 111, 97, 83, 71, **57**, 43
96	20.4	octacosane	125, 111, 97, 83, 71, **57**, 43
97	20.6	hexacosanal	362, 123, 109, 96, 82, 69, **57**, 43
98	20.9	nonacosane	139, 125, 111, 97, 83, 69, **57**, 43
99	21.1	heptacosanal	376, 123, 111, 96, 82, 71, **57**, 43
100	21.4	triacontane	139, 125, 111, 97, 83, 71, **57**, 43
101	22.0	hentriacontane	139, 125, 111, 97, 83, 71, **57**, 43
102	22.7	dotriacontane	125, 111, 97, 85, 71, **57**, 43

Table S.6 Identification of peaks in the chromatogram obtained in the Py-GC-MS analysis of HDPE-0w.

#	t_r	Peak identification	Main ions (*m*/*z*)
1	2.7	1-hexene	84, 69, 56, **41**
2	4.0	1-heptene	98, 83, 70, **56**, 41
3	4.1	heptane	100, 71, 57, **43**
4	6.8	1-octene	112, 97, 83, 70, **55**, 41
5	7.0	octane	114, 85, 71, 57, **43**
6	8.7	1,8-nonadiene	109, 96, 81, **67**, 55, 41
7	8.9	1-nonene	126, 97, 83, 69, **56**, 41
8	9.0	nonane	128, 99, 85, 71, 57, **43**
9	10.2	1,9-decadiene	110, 95, 81, 67, **55**, 41
10	10.3	1-decene	140, 111, 97, 83, 70, 55, **41**
11	10.4	decane	142, 117, 99, 85, 71, **57**, 43
12	11.2	1,10-undecadiene	124, 109, 95, 81, 67, 55, **41**
13	11.3	1-undecene	154, 126, 111, 97, 83, 70, 55, **43**
14	11.4	undecane	156, 98, 85, 71, **57**, 43
15	12.1	1,11-dodecadiene	138, 124, 109, 95, 81, 67, **55**, 41
16	12.16	1-dodecene	168, 140, 125, 111, 97, 83, 69, 55, **41**
17	12.2	dodecane	170, 128, 85, 71, **57**, 43
18	12.3	decanal	138, 128, 112, 95, 82, 68, 55, **41**
19	12.87	1,12-tridecadiene	123, 109, 95, 81, 67, **55**, 41
20	12.9	1-tridecene	125, 111, 97, 83, 69, **55**, 41
21	13.0	tridecane	184, 99, 85, 71, **57**, 43
22	13.05	undecanal	152, 126, 109, 96, 82, 68, **57**, 41
23	13.5	1,13-tetradecadiene	123, 109, 96, 81, 67, **55**, 41
24	13.6	1-tetradecene	125, 111, 97, 83, 69, **55**, 41
25	13.7	tetradecane	198, 99, 85, 71, **57**, 43
26	13.75	dodecanal	166, 140, 123, 110, 96, 82, 68, **57**, 41
27	14.2	1,14-pentadecadiene	123, 109, 96, 81, 67, **55**, 41
28	14.27	1-pentadecene	210, 125, 111, 97, 83, 69, **55**, 41
29	14.3	pentadecane	212, 113, 99, 85, 71, **57**, 43
30	14.4	tridecanal	180, 154, 124, 110, 96, 82, 68, **57**, 43
31	14.8	1,15-hexadecadiene	123, 109, 96, 82, 67, **55**, 41
32	14.88	1-hexadecene	224, 125, 111, 97, 83, 69, 55, **41**
33	14.9	hexadecane	226, 99, 85, 71, **57**, 43

34	15.0	tetradecanal	194, 168, 138, 110, 96, 82, 69, **57**, 41
35	15.4	1,16-heptadecadiene	137, 123, 109, 96, 82, 69, **55**, 41
36	15.46	1-heptadecene	238, 139, 125, 111, 97, 83, 69, **55**, 41
37	15.5	heptadecane	240, 99, 85, 71, **57**, 43
38	15.6	pentadecanal	208, 180, 110, 96, 82, 69, **57**, 41
39	15.9	1,17-octadecadiene	123, 109, 96, 82, 69, **55**, 41
40	16.0	1-octadecene	252, 125, 111, 97, 83, 69, **55**, 41
41	16.04	octadecane	254, 99, 85, 71, **57**, 43
42	16.1	hexadecanal	222, 123, 109, 96, 82, 69, **57**, 43
43	16.5	1,18-nonadecadiene	137, 123, 109, 96, 82, 69, **55**, 41
44	16.54	1-nonadecene	139, 125, 111, 97, 83, 69, **55**, 43
45	16.6	nonadecane	268, 127, 113, 99, 85, 71, **57**, 43
46	16.7	heptadecanal	236, 208, 137, 123, 110, 96, **82**, 68, 57, 41
47	17.0	1,19-eicosadiene	137, 123, 109, 96, 82, 69, **55**, 41
48	17.03	1-eicosene	139, 125, 111, **97**, 83, 69, 55, 43
49	17.06	eicosane	127, 113, 99, 85, 71, **57**, 43
50	17.2	octadecanal	250, 137, 124, 109, 96, **82**, 68, 57, 43
51	17.4	1,20-heneicosadiene	137, 123, 109, 96, 82, 69, **55**, 41
52	17.5	1-heneicosene	139, 125, 111, **97**, 83, 69, 55, 43
53	17.53	heneicosane	113, 99, 85, 71, **57**, 43
54	17.7	nonadecanal	264, 202, 137, 124, 110, 96, **82**, 68, 57, 43
55	17.94	1,21-docosadiene	149, 137, 123, 109, 96, 82, 69, **55**, 41
56	17.96	1-docosene	139, 125, 111, **97**, 83, 69, 55, 43
57	17.98	docosane	310, 97, 85, 71, **57**, 43
58	18.1	eicosanal	278, 250, 124, 109, 96, **82**, 68, 57, 43
59	18.3	1,22-tricosadiene	137, 123, 109, 96, 82, 69, **55**, 41
60	18.40	1-tricosene	322, 125, 111, **97**, 83, 69, 55, 43
61	18.41	tricosane	324, 111, 97, 85, 71, **57**, 43
62	18.5	heneicosanal	292, 138, 123, 110, 96, **82**, 68, 57, 43
63	18.8	1,23-tetracosadiene	334, 137, 123, 109, 96, 82, 69, **55**, 41
64	18.81	1-tetracosene	336, 139, 125, 111, **97**, 83, 69, 55, 43
65	19.0	docosanal	306, 251, 138, 123, 110, 96, **82**, 68, 57, 43
66	19.2	1-pentacosene	350, 139, 125, 111, **97**, 83, 69, 57, 43
67	19.4	tricosanal	320, 138, 124, 110, 96, **82**, 68, 57, 43
68	19.6	1,25-hexacosadiene	362, 139, 125, 111, 97, 83, 69, **57**, 43
69	19.8	unknown	**297**, 149, 105, 77

Table S.7 Identification of peaks in the chromatogram obtained in the Py-GC-MS analysis of HDPE-4w.

#	t_r	Peak identification	Main ions (*m*/*z*)
1	2.7	1-hexene	84, 69, 56, **41**
2	4.0	1-heptene	98, 83, 70, **56**, 41
3	4.1	heptane	100, 71, 57, **43**
4	6.7	1-octene	112, 97, 83, 70, **55**, 41
5	6.9	octane	114, 85, 71, 57, **43**
6	8.7	1,8-nonadiene	109, 96, 81, **67**, 55, 41
7	8.9	1-nonene	126, 97, 83, 69, **56**, 41
8	9.0	nonane	128, 99, 85, 71, 57, **43**
9	10.1	1,9-decadiene	110, 95, 81, 67, **55**, 41
10	10.2	1-decene	140, 111, 97, 83, 70, 55, **41**

11	10.34	decane	142, 113, 99, 85, 71, **57**, 43
12	11.2	1,10-undecadiene	124, 109, 95, 81, 67, 55, **41**
13	11.3	1-undecene	154, 126, 111, 97, 83, 70, **55**, 41
14	11.36	undecane	156, 98, 85, 71, **57**, 43
15	11.4	nonanal	124, 114, 98, 82, 70, **57**, 41
16	12.1	1,11-dodecadiene	138, 124, 109, 95, 81, 67, **55**, 41
17	12.15	1-dodecene	168, 140, 125, 111, 97, 83, 69, 55, **41**
18	12.2	dodecane	170, 128, 85, 71, **57**, 43
19	12.3	decanal	138, 128, 112, 95, 82, 70, 57, **41**
20	12.7	nonanoic acid	158, 129, 115, 98, **85**, 73, 60, 41
21	12.8	1,12-tridecadiene	123, 109, 95, 81, 67, **55**, 41
22	12.9	1-tridecene	125, 111, 97, 83, 69, 55, **41**
23	13.0	tridecane	184, 99, 85, 71, **57**, 43
24	13.04	undecanal	142, 126, 109, 96, 82, 68, 57, **41**
25	13.4	decanoic acid	172, 129, **73**, 60, 41
26	13.48	undecanol	126, 111, 97, **85**, 69, 55, 41
27	13.5	1,13-tetradecadiene	123, 109, 95, 81, 67, **55**, 41
28	13.6	1-tetradecene	125, 111, 97, 83, 69, **55**, 41
29	13.66	tetradecane	198, 99, 85, 71, **57**, 43
30	13.7	dodecanal	166, 140, 123, 110, 96, 82, 68, **57**, 41
31	14.06	undecanoic acid	186, 143, 129, 115, 85, **73**, 60, 41
32	14.1	dodecanol	141, 127, 111, 97, 83, 69, **55**, 43
33	14.2	1,14-pentadecadiene	123, 109, 96, 81, 67, **55**, 41
34	14.26	1-pentadecene	210, 125, 111, 97, 83, 69, **55**, 41
35	14.3	pentadecane	141, 127, 113, 99, 85, 71, **57**, 43
36	14.4	tridecanal	180, 154, 124, 110, 96, 82, 69, **57**, 41
37	14.6	dodecanoic acid	200, 157, 129, 115, 101, 85, **73**, 60, 43
38	14.77	tridecanol	182, 155, 141, 125, 111, 97, 83, 69, 55, **43**
39	14.8	1,15-hexadecadiene	123, 109, 96, 82, 67, **55**, 41
40	14.88	1-hexadecene	224, 125, 111, 97, 83, 69, **55**, 41
41	14.9	hexadecane	226, 99, 85, 71, **57**, 43
42	15.0	tetradecanal	194, 168, 138, 124, 109, 96, 82, 69, **57**, 41
43	15.2	tridecanoic acid	214, 185, 171, 157, 143, 129, 115, 87, **73**, 60
44	15.36	tetradecanol	169, 125, 111, 97, 83, 69, **55**, 43
45	15.4	1,16-heptadecadiene	137, 123, 109, 96, 82, 69, **55**, 41
46	15.45	1-heptadecene	238, 139, 125, 111, 97, 83, 69, **55**, 41
47	15.5	heptadecane	240, 99, 85, 71, **57**, 43
48	15.6	pentadecanal	208, 182, 109, 96, 82, 69, **57**, 41
49	15.8	tetradecanoic acid	228, 185, 129, **73**
50	15.93	pentadecanol	183, 125, 111, 97, 83, 69, **57**, 41
51	15.96	1,17-octadecadiene	123, 109, 96, 82, 69, **55**, 41
52	16.0	1-octadecene	252, 125, 111, 97, 83, 69, **55**, 41
53	16.04	octadecane	254, 99, 85, 71, **57**, 43
54	16.06	2-hexadecanone	240, 111, 97, 85, 71, **58**, 43
55	16.1	hexadecanal	222, 123, 109, 96, 82, 69, **57**, 41
56	16.3	pentadecanoic acid	242, 199, 129, **73**, 60
57	16.46	hexadecanol	197, 125, 111, 97, 83, 69, **55**, 43
58	16.5	1,18-nonadecadiene	137, 123, 109, 96, 82, 69, **55**, 41
59	16.53	1-nonadecene	139, 125, 111, 97, 83, 69, **55**, 43
60	16.55	nonadecane	268, 127, 113, 99, 85, 71, **57**, 43
61	16.58	2-heptadecanone	254, 85, 71, **58**, 43
62	16.7	heptadecanal	236, 208, 137, 123, 109, 96, **82**, 69, 57, 43

63	16.8	hexadecanoic acid	256, 213, 129, **73**, 60
64	16.99	1,19-eicosadiene	137, 123, 109, 96, 82, 69, **55**, 41
65	17.0	1-eicosene	139, 125, 111, **97**, 83, 69, 55, 43
66	17.04	eicosane	127, 113, 99, 85, 71, **57**, 43
67	17.09	2-octadecanone	268, 109, 96, 85, **71**, 58, 43
68	17.2	octadecanal	250, 137, 123, 109, 96, 82, 69, **57**, 43
69	17.3	heptadecanoic acid	270, 227, 185, 171, 129, **73**, 60
70	17.4	1,20-heneicosadiene	137, 123, 109, 96, 82, 69, **55**, 41
71	17.5	1-heneicosene	139, 125, 111, **97**, 83, 69, 55, 43
72	17.52	heneicosane	111, 97, 85, 71, **57**, 43
73	17.56	2-nonadecanone	282, 127, 111, 97, 85, 71, **58**, 43
74	17.7	nonadecanal	264, 236, 137, 123, 109, 96, **82**, 69, 57, 43
75	17.8	octadecanoic acid	284, 241, 185, 129, **73**, 60
76	17.92	1,21-docosadiene	137, 123, 109, 96, 82, 69, **55**, 41
77	17.95	1-docosene	139, 125, 111, **97**, 83, 69, 55, 43
78	17.97	docosane	310, 97, 85, 71, **57**, 43
79	18.02	2-eicosanone	297, 127, 96, 85, 71, **58**, 43
80	18.1	eicosanal	278, 250, 123, 109, 96, **82**, 69, 57, 43
81	18.2	nonadecanoic acid	298, 255, 129, 85, **73**
82	18.37	1,22-tricosadiene	137, 123, 111, 97, 83, 69, **55**, 41
83	18.39	1-tricosene	322, 125, 111, **97**, 83, 69, 57, 43
84	18.40	tricosane	324, 111, 97, 85, 71, **57**, 43
85	18.46	2-heneicosanone	310, 124, 109, 96, 85, 71, 58, **43**
86	18.5	heneicosanal	292, 137, 123, 111, 96, **82**, 69, **57**, 43
87	18.78	1,23-tetracosadiene	334, 137, 123, 111, 97, 83, 69, **55**, 43
88	18.8	1-tetracosene	336, 139, 125, 111, **97**, 83, 69, 57, 43
89	18.9	2-docosanone	324, 309, 109, 96, 82, 71, **59**, 43
90	19.0	docosanal	306, 250, 137, 125, 111, 97, 82, 71, **57**, 43
91	19.2	1-pentacosene	350, 139, 125, 111, 97, 83, 69, **57**, 43
92	19.4	tricosanal	320, 137, 123, 111, 97, 82, 69, **57**, 43
93	19.6	1,25-hexacosadiene	362, 139, 125, 111, 97, 83, 69, **55**, 43
94	19.7	tetracosanal	334, 138, 123, 109, 96, **82**, 68, 57, 43
95	20.0	heptacosane	380, 125, 111, 97, 85, 71, **57**, 43
96	20.2	pentacosanal	348, 123, 111, 97, 83, 69, **57**, 43
97	20.4	octacosane	125, 111, 97, 85, 71, **57**, 43
98	20.6	hexacosanal	362, 123, 111, 96, 82, 69, **57**, 43
99	20.9	nonacosane	139, 125, 111, 97, 83, 71, **57**, 43
100	21.1	heptacosanal	376, 123, 111, 96, 82, 71, **57**, 43
101	21.4	triacontane	139, 125, 111, 97, 83, 71, **57**, 43

Table S.8 List of the main pyrolysis products in the chromatogram obtained in the Py(HMDS)-GC-MS analysis of the DCM extract of PP-0w (**Figure S.12**).

#	t_r	Peak identification	Main ions (*m*/*z*)
1	13.2	2,4-dimethyl-1-heptene	165, 149, 126, 83, **70**, 55
2	16.5	HMDS unknown	**222**, 206, 190, 176, 132, 74
3	17.2	hexamethyl-cyclotrisiloxane	**207**, 191, 96
4	17.8	octamethyl-cyclotetrasiloxane	**281**, 265, 149, 191, 133, 73
5	17.9	p- tolypentamethyl-disiloxane	238, **223**, 207, 149, 104, 73
6	18.4	decamethyl-tetrasiloxane	295, **207**, 73

7	18.5	unknown	**236**, 204, 190, 132, 73
8	18.7	unkonown	177, **162**, 134, 100, 73
9	19.0	unknown	222, 206, 193, **130**, 116, 100, 73
10	19.2	unknown	280, **266**, 192, 125
11	20.1	2,2,3,3,7,7,8,8-octamethyl-4,6-dioxa-5-aza-2,3,7,8-tetrasilanonane	294, **206**, 190, 73
12	21.4	unknown	**293**, 205, 146, 130, 73
13	23.4	tetradecamethyl-hexasiloxane	443, 355, 281, 267, 221, 147, **73**
14	24.5	decanoic acid, trimethylsilyl ester	244, **229**, 145, 129, 117, 73
15	24.7	tetradecamehylcycloheptasiloxane	503, 415, 341, 327, 281, 147, **73**
16	25.2	1-cyclohexyldimethylsilyloxyundec-2-ene	**227**, 143, 129, 75

Table S.9 List of the main pyrolysis products in the chromatogram obtained in the Py(HMDS)-GC-MS analysis of the MeOH extract of PS-0w (**Figure S.13**).

#	t_r	Peak identification	Main ions (*m/z*)
1	14.8	ethylbenzene	106, **91**, 77, 65, 51
2	15.4	styrene	**104**, 89, 78, 63, 51
3	15.5	silanamine, 1,1,1-trimethyl-N-(trimethylsilyl)-	**146**, 130, 100, 73, 59
4	16.0	benzene, (1-methylethyl)-	120, **105**, 91, 77, 51
5	16.6	unknown	175, 146, **132**, 115, 102
6	16.7	HMDS unknown	**222**, 206, 190, 132, 74
7	17.3	HMDS unknown	**220**, 207, 132, 73
8	17.4	cyclotrisiloxane, hexamethyl-	**207**, 191, 133, 96
9	17.9	cyclotetrasiloxane, octamethyl-	**281**, 265, 207, 191, 133, 73
10	18.5	tetrasiloxane, decamethyl-	295, **207**, 191, 73
11	18.6	silane, trimethylphenoxy-	166, **151**, 135, 91, 77
12	18.8	propanoic acid, 2-[(trimethylsilyl)oxy]-, trimethylsilyl ester	191, **147**, 133, 117, 73
13	19.9	cyclotetrasiloxane, octamethyl-	**281**, 265, 249, 133, 73
14	20.1	4,6-dioxa-5-aza-2,3,7,8-tetrasilanonane-2,2,3,3,7,7,8,8-octamethyl-	294, **206**, 190, 130, 73
15	20.9	cyclopentasiloxane, decamethyl	355, 267, 251, 187, **73**
16	21.3	pentasiloxane, dodecamethyl-	369, 353, **281**, 265, 249, 207, 147, 43
17	21.4	unknown	**293**, 205, 189, 146, 130, 73
18	22.7	cyclohexasiloxane, dodecamethyl-	429, 341, 207, 147, **73**
19	23.4	hexasiloxane, tetradecamethyl-	443, 355, 281, 267, 221, 147, **73**
20	25.2	HMDS unknown	265, 250, **146**, 132, 73
21	25.9	propane-1,2-diyldibenzen	196, 115, **105**, 91, 77
22	26.4	1-pentene-2,4-diyldibenzen	165, **105**, 91, 77
23	26.7	benzene, 1,1'-(1,2-dimethyl-1,2-ethanediyl)bis-	210, **105**, 91, 77
24	27.8	3-butene-1,3-diyldibenzene (styrene dimer)	208, 193, 130, 115, 104, **91**, 77, 65
25	28.4	1H-indene, 2-phenyl-	**192**, 165, 115, 91
26	28.5	naphthalene, 1,2,3,4-tetrahydro-1-phenyl-	**208**, 191, 180, 165, 152, 130, 115, 91
27	28.7	naphthalene, 1,2-dihydro-4-phenyl-	**206**, 191, 128, 115, 91
28	28.8	anthracene	**178**, 152, 89, 76
29	29.9	unknown	299, 281, 237, **163**, 135, 103, 73
30	30.4	fluoranthene, 1,2,3,10b-tetrahydro-	206, 190, **178**, 165, 152, 89, 76
31	30.7	unknown	161, 147, 117, 103, **73**
32	30.9	hexadecanoic acid, trimethylsilyl ester	328, 313, 145, 129, 117, 73
33	32.6	octadecanoic acid, trimethylsilyl ester	341, 145, 129, 117, **73**
34	33.0	uknown	161, 147, 133, 117, 103, 87, **73**

35	34.6	5-hexene-1,3,5-triyltribenzene (styrene trimer)	312, 207, 194, 117, **91**, 77

Table S.10 List of the main pyrolysis products in the chromatogram obtained in the Py(HMDS)-GC-MS analysis of the DCM extract of LDPE-4w (**Figure 14**).

#	t_r	Peak identification	Main ions (*m*/*z*)
1	15.2	butanoic acid, trimethylsilyl ester	145, 117, **75**
2	15.4	silanamine, 1,1,1-trimethyl-N-(trimethylsilyl)-	**146**, 130, 100, 73, 59
3	16.2	2-butenoic acid, trimethylsilyl ester	**143**, 99, 75
4	16.5	unknown	175, 146, **132**, 115, 73
5	16.6	HMDS unknown	**222**, 206, 190, 176, 132, 74
6	16.7	HMDS unknown	237, **222**, 206, 190, 176, 132, 74
7	17.0	4-pentenoic acid, trimethylsilyl ester	157, 117, **75**
8	17.2	pentanoic acid, trimethylsilyl ester	159, 145, 132, 117, **75**
9	17.3	HMDS unknown	**220**, 207, 132, 73
10	17.4	hexamethyl-cyclotrisiloxane	**207**, 191, 96
11	17.8	3-butenoic, -methyl-, trimethylsilyl ester	172, 157, 113, 82, **75**
12	17.9	cyclotetrasiloxane, octamethyl-	**281**, 265, 249, 207, 191, 133, 73
13	18.5	tetrasiloxane, decamethyl-	295, **207**, 191, 73
14	18.7	hexenoic acid, trimethylsilyl ester	171, 129, 117, **75**
15	18.8	propanoic acid, 2-[(trimethylsilyl)oxy]-, trimethylsilyl ester	191, **147**, 133, 117, 73
16	19.0	hexanoic acid, trimethylsilyl ester	173, 145, 131, 117, **75**
17	19.1	acetic acid, [(trimethylsilyl)oxy]-, trimethylsilyl ester	205, 177, **147**, 133, 73
18	19.4	undecane	156, 113, 98, 85, 71, **57**
19	20.0	pentanoic acid, 4-oxo, trimethylsilyl ester	173, 145, 131, **75**
20	20.1	4,6-dioxa-5-aza-2,3,7,8-tetrasilanonane-2,2,3,3,7,7,8,8-octamethyl-	294, **206**, 190, 130, 73
21	20.2	propanoic acid, 3-[(trimethylsilyl)oxy]-, trimethylsilyl ester	219, 177, **147**, 133, 116, 73
22	20.3	heptenoic acid, trimethylsilyl ester	185, 129, 117, **75**
24	20.5	heptanoic acid, trimethylsilyl ester	187, 131, 117, **75**
25	20.7	pentasiloxane, dodecamethyl-	369, 281, 207, 147, **73**
26	20.8	1-dodecene	168, 111, 97, 83, 69, **55**
27	21.3	pentasiloxane, dodecamethyl-	369, 353, **281**, 265, 249, 207, 147, 43
28	21.4	unknown	**293**, 277, 205, 189, 146, 130,
29	21.7	benzoic acid, trimethylsilyl ester	194, **179**, 135, 105, 77, 51
30	21.8	octenoic acid, trimethylsilyl ester	199, 129, 117, **75**
31	21.9	octanoic acid, trimethylsilyl ester	201, 145, 129, 117, **75**
32	22.3	1-tridecene	182, 154, 125, 111, 97, 83, 69, **55**
33	22.7	butanedioic acid, bis(trimethylsilyl ester)	247, 172, **147**, 129, 73
34	22.8	butanedioic acid, methyl-, bis(trimethylsilyl) ester	261, 232, 217, 186, **147**, 73
35	23.2	cyclohexasiloxane, dodecamethyl-	429, 341, 147, **73**
36	23.2	nonenoic acid, trimethylsilyl ester	**215**, 145, 129, 117, 73
37	23.3	nonanoic acid, trimethylsilyl ester	230, **215**, 145, 129, 117, 73
38	23.4	hexasiloxane, tetradecamethyl-	443, 355, 281, 267, 221, 147, **73**
39	23.7	1-tetradecene	196, 141, 125, 111, 97, 83, 69, **55**
40	23.9	pentanedioic acid, bis(trimethylsilyl) ester	261, 233, 204, 158, **147**, 129, 116, 97, 73
41	24.4	decenoic acid, trimethylsilyl ester	227, 145, 129, 117, **75**
42	24.5	decanoic acid, trimethylsilyl ester	**229**, 145, 129, 117, 73

43	24.9	1-pentadecene	210, 140, 125, 111, 83, 69, **55**
44	25.0	pentadecane	212, 141, 127, 113, 99, 85, 71, **57**
45	25.2	hexanedioic acid, bis(trimethylsilyl) ester	275, 217, 204, 172, 147, 129, 111, **73**
46	25.6	undecenoic acid, trimethylsilyl ester	241, 145, 129, 117, 96, **75**
47	25.7	undecanoic acid, trimethylsilyl ester	**243**, 145, 129, 117, 95, 73
48	26.2	1-hexadecene	224, 125, 111, 97, 83, 69, **55**
49	26.3	hexadecane	226, 127, 113, 99, 85, 71, **57**
50	26.4	heptanedioic acid, bis(trimethylsilyl) ester	289, 217, 204, 186, 155, 147, 125, 97, **73**
51	26.8	dodecenoic acid, trimethylsilyl ester	255, 201, 145, 129, 117, **73**
52	26.9	dodecanoic acid, trimethylsilyl ester	**257**, 201, 145, 129, 117, 73
53	27.3	1-heptadecene	238, 125, 111, 97, 83, 69, **55**
54	27.4	octanedioic acid, bis(trimethylsilyl) ester	303, 217, 187, 169, 147, 129, 117, **73**
55	27.8	tridecenoic acid, trimethylsilyl ester	269, 145, 129, 117, **73**
56	27.9	tridecanoic acid, trimethylsilyl ester	271, 145, 129, 117, **73**
57	28.4	1-octadecene	252, 139, 125, 111, 97, 83, 69, **55**
58	28.5	nonanedioic acid, bis(trimethylsilyl) ester	317, 217, 201, 147, 129, 117, 97, **73**
59	28.9	tetradecenoic acid, trimethylsilyl ester	283, 145, 129, 117, **73**
60	29.0	tetradecanoic acid, trimethylsilyl ester	300, **285**, 145, 132, 117, 73
61	29.4	1-nonadecene	266, 139, 125, 111, 97, 83, 69, **55**
62	29.5	decanedioic acid, bis(trimethylsilyl) ester	331, 315, 215, 204, 185, 147, 129, 117, **73**
63	29.8	pentadecenoic acid, trimethylsilyl ester	297, 145, 129, 117, **73**
64	29.9	pentadecanoic acid, trimethylsilyl ester	299, 145, 129, 117,**73**
65	30.4	eicosane	282, 127, 113, 99, 85, 71, **57**
66	30.5	undecanedioic acid, bis(trimethylsilyl) ester	345, 317, 129, 117, **73**
67	30.8	hexadecenoic acid, trimethylsilyl ester	311, 145, 129, 117, **73**
68	30.9	hexadecanoic acid, trimethylsilyl ester	**313**, 145, 129, 117, 73
69	31.2	1,20-heneicosadiene	123, 109, 96, 82, 69, **55**
70	31.3	1-heneicosene	139, 125, 111, **97**, 83, 69, 55
71	31.4	heneicosane	296, 127, 113, 99, 85, 71, **57**
72	31.7	heptadecenoic acid, trimethylsilyl ester	325, 145, 129, 117, **73**
73	31.8	heptadecanoic acid, trimethylsilyl ester	342, **327**, 201, 145, 132, 117, 73
74	32.1	1,21-docosadiene	306, 123, 109, 96, 82, 69, **55**
75	32.2	1-docosene	139, 125, 111, **97**, 83, 69, 55
76	32.3	docosane	127, 113, 99, 85, 71, **57**
77	32.5	2,2-bis[(4-trimethylsiloxy)phenyl]propane	372, **357**, 207, 73
78	32.6	octadecenoic acid, trimethylsilyl ester	339, 145, 129, **117**, 97, 75
79	32.7	octadecanoic acid, trimethylsilyl ester	341, 145, 129, 117, **73**
80	33.0	1,22-tricosadiene	137, 123, 109, 96, 82, 69, **55**
81	33.06	1-tricosene	322, 125, 111, **97**, 83, 69, 55
82	33.1	tricosane	324, 113, 97, 85, 71, **57**
83	33.4	nonadecenoic acid, trimethylsilyl ester	**353**, 145, 129, 117, 97, 75
84	33.5	nonadecanoic acid, trimethylsilyl ester	355, 145, 132, **117**, 97, 73
85	33.8	1,23-tetracosadiene	334, 137, 123, 109, 96, 83, 69, **55**
86	33.9	1-tetracosene	336, 139, 125, 111, 97, 83, 69, **57**
87	33.91	tetracosane	338, 127, 113, 99, 85, 71, **57**
88	34.2	eicosenoic acid, trimethylsilyl ester	367, 145, 129, 117, 97, **73**
89	34.3	eicosanoic acid, trimethylsilyl ester	384, **369**, 341, 201, 145, 132, 117, 73
90	34.5	hexanedecandioic acid, bis(trimethylsilyl) ester	415, 229, 217, 204, 129, 117, **73**
91	34.6	1,24-pentacosadiene	137, 123, 109, 96, 82, 69, **55**
92	34.7	1-pentacosene	139, 125, 111, **97**, 83, 69, 57
93	34.9	heneicosenoic acid, trimethylsilyl ester	381, 145, 129, 117, **73**
94	35.0	heneicosanoic acid, trimethylsilyl ester	398, 383, 145, 132, **117**, 73

95	35.3	heptanedecanedioic acid, bis(trimethylsilyl) ester	429, 313, 217, 204, 129, 117, **73**
96	35.4	1-hexacosene	139, 125, 111, 97, 83, 69, **57**
97	35.7	docosenoic acid, trimethylsilyl ester	395, 145, 129, 117, **73**
98	35.8	docosanoic acid, trimethylsilyl ester	412, 397, 145, 129, 117, 97, **73**
99	36.0	octanedecanedioic acid, bis(trimethylsilyl) ester	443, 327, 217, 204, 129, 117, **73**
100	36.1	1-heptacosene	139, 125, 111, **97**, 83, 69, 57
101	36.4	tricosenoic acid, trimethylsilyl ester	409, 145, 129, 117, 97, **73**
102	36.5	tricosanoic acid, trimethylsilyl ester	411, 145, 129, 117, **73**
103	36.9	1-octacosene	139, 125, 111, 97, 83, 69, **57**
104	37.2	tetracosanoic acid, trimethylsilyl ester	425, 145, 129, 117, 97, **73**
105	37.7	1-nonacosene	139, 125, 111, 97, 83, 69, **57**
106	38.0	pentacosanoic acid, trimethylsilyl ester	454, 439, 145, 132, **117**, 73
107	38.5	1-triacontene	139, 125, 111, 97, 83, 69, **57**
108	38.9	hexacosanoic acid, trimethylsilyl ester	468, 453, 145, 132, **117**, 73
109	39.6	1-hentriacontene	139, 125, 111, 97, 83, 71, **57**
110	40.0	heptacosanoic acid, trimethylsilyl ester	482, 467, 369, 145, 129, **117**, 73
111	40.7	1-dotriacontene	139, 125, 111, 97, 83, 71, **57**
112	41.3	octacosanic acid, trimethylsilyl ester	496, 481, 145, 129, 117, 97, **73**
113	42.2	1-tritriacontene	139, 125, 111, 97, 83, 69, **57**
114	42.8	nonacosanoic acid, trimethylsilyl ester	510, 495, 145, 132, **117**, 73
115	43.9	1-tetratriacontene	139, 125, 111, 97, 83, 71, **57**
116	44.6	triacontanoic acid, trimethylsilyl acid	524, 509, 145, 132, **117**, 97, 73
117	45.9	1-pentatriacontene	139, 125, 111, 97, 83, 71, **57**
118	46.8	hentriacontanoic acid, trimethylsilyl ester	538, 523, 145, 132, 117, 97, **73**
119	47.9	1-hexatriacontene	139, 125, 111, 97, 83, 71, **57**
120	48.7	dotriacontanoic acid, trimethylsilyl ester	552, 537, 145, 129, 117, **73**
121	50.9	tritriacontanoic acid, trimethylsilyl ester	556, 551, 145, 132, 117, **73**
122	53.5	tetratriacontanoic acid, trimethylsilyl ester	580, 565, 145, 132, 117, **73**
123	56.7	pentatriacontanoic acid, trimethylsilyl ester	594, 579, 145, 132, 117, **73**

Table S.11 List of the main pyrolysis products in the chromatogram obtained in the Py(HMDS)-GC-MS analysis of the DCM extract of LDPE-0w (**Figure S.15**).

#	t_r	Peak identification	Main ions (*m/z*)
1	13.4	unknown	**164**, 134, 118, 74
2	14.7	silanamine, 1,1,1-trimethyl-N-(trimethylsilyl)-	**146**, 130, 100, 73, 59
3	16.3	HMDS unknown	**222**, 206, 190, 176, 132, 74
4	16.4	HMDS unknown	**220**, 206, 190, 132, 74
5	17.0	hexamethyl-cyclotrisiloxane	**207**, 191, 96
6	17.1	HMDS unknown	**220**, 204, 132, 73
7	17.2	HMDS unknown	223, **207**, 191, 133
8	18.4	decamethyltetrasiloxane	295, **207**, 73
9	18.5	HMDS unknown	**236**, 204, 190, 132, 73
10	18.9	HMDS unknown	222, **206**, 190, 132, 74
11	20.1	2,2,3,3,7,7,8,8-octamethyl-4,6-dioxa-5-aza-2,3,7,8-tetrasilanonane	294, **206**, 190, 73
12	21.5	HMDS unknown	**293**, 205, 146, 130, 73
13	23.2	dodecamethylcyclohexasiloxane	429, 341, 325, 147, **73**
14	24.3	HMDS unknown	428, 340, 324, 206, 146, **73**
15	24.4	unknown	231, 216, **146**, 130, 73
16	24.5	HMDS unknown	428, 340, 324, 206, 146, **73**

17	24.7	tetradecamethylcycloheptasiloxane	503, 415, 341, 327, 281, 147, **73**
18	26.3	hexadecane	226, 113, 99, 85, 71, **57**
19	28.5	octadecane	254, 113, 99, 85, 71, **57**
20	30.5	eicosane	282, 113, 99, 85, 71, **57**
21	32.3	docosane	310, 113, 99, 85, 71, **57**
22	33.9	tetracosane	338, 113, 99, 85, 71, **57**
23	35.5	hexacosane	366, 113, 99, 85, 71, **57**
24	36.9	octacosane	127, 113, 99, 85, 71, **57**
25	38.6	triacontane	127, 113, 99, 85, 71, **57**

Table S.12 List of the main pyrolysis products in the chromatogram obtained in the Py(HMDS)-GC-MS analysis of the DCM extract of HDPE-0w.

#	t_r	Peak identification	Main ions (*m*/*z*)
1	13.4	unknown	**164**, 134, 118, 74
2	14.7	silanamine, 1,1,1-trimethyl-N-(trimethylsilyl)-	**146**, 130, 100, 73, 59
3	16.3	HMDS unknown	**222**, 206, 190, 176, 132, 74
4	16.4	HMDS unknown	**220**, 206, 190, 132, 74
5	17.0	hexamethyl-cyclotrisiloxane	**207**, 191, 96
6	17.1	HMDS unknown	**220**, 204, 132, 73
7	17.2	HMDS unknown	223, **207**, 191, 133
8	18.4	decamethyltetrasiloxane	295, **207**, 73
9	18.5	HMDS unknown	**236**, 204, 190, 132, 73
10	18.9	HMDS unknown	222, **206**, 190, 132, 74
11	20.1	2,2,3,3,7,7,8,8-octamethyl-4,6-dioxa-5-aza-2,3,7,8-tetrasilanonane	294, **206**, 190, 73
12	21.5	HMDS unknown	**293**, 205, 146, 130, 73
13	23.2	dodecamethylcyclohexasiloxane	429, 341, 325, 147, **73**
14	24.3	HMDS unknown	428, 340, 324, 206, 146, **73**
15	24.4	unknown	231, 216, **146**, 130, 73
16	24.5	HMDS unknown	428, 340, 324, 206, 146, **73**
17	24.7	tetradecamethylcycloheptasiloxane	503, 415, 341, 327, 281, 147, **73**
18	26.3	hexadecane	226, 113, 99, 85, 71, **57**
19	28.5	octadecane	254, 113, 99, 85, 71, **57**
20	30.5	eicosane	282, 113, 99, 85, 71, **57**
21	32.3	docosane	310, 113, 99, 85, 71, **57**
22	33.9	tetracosane	338, 113, 99, 85, 71, **57**
23	35.5	hexacosane	366, 113, 99, 85, 71, **57**
24	36.9	octacosane	127, 113, 99, 85, 71, **57**
25	38.6	triacontane	127, 113, 99, 85, 71, **57**

Table S.13 List of the main pyrolysis products in the chromatogram obtained in the Py(HMDS)-GC-MS analysis of the DCM extract of HDPE-4w.

#	t_r	Peak identification	Main ions (*m*/*z*)
1	15.2	3-butenoic acid, trimethylsilyl ester	143, 117, 99, **73**
2	15.5	silanamine, 1,1,1-trimethyl-N-(trimethylsilyl)-	**146**, 130, 100, 73, 59
3	16.3	2-butenoic acid, trimethylsilyl ester	**143**, 99, 75

4	16.6	unknown	175, 146, **132**, 115, 73
5	16.7	HMDS unknown	**222**, 206, 190, 176, 132, 74
6	17.0	4-pentenoic acid, trimethylsilyl ester	157, 117, **75**
7	17.2	pentanoic acid, trimethylsilyl ester	159, 145, 132, 117, **75**
8	17.3	HMDS unknown	**220**, 207, 132, 73
9	17.4	hexamethyl-cyclotrisiloxane	**207**, 191, 96
10	17.44	1-decene	140, 111, 97, 83, 70, **55**
11	17.9	cyclotetrasiloxane, octamethyl-	**281**, 265, 207, 191, 133, 73
12	18.5	tetrasiloxane, decamethyl-	295, **207**, 191, 73
13	18.8	hexenoic acid, trimethylsilyl ester	171, 129, 117, **75**
14	19.0	hexanoic acid, trimethylsilyl ester	173, 117, **75**
15	20.0	pentanoic acid, 4-oxo, trimethylsilyl ester	173, 145, 131, **75**
16	20.1	4,6-dioxa-5-aza-2,3,7,8-tetrasilanonane-2,2,3,3,7,7,8,8-octamethyl-	294, **206**, 190, 130, 73
17	20.2	propanoic acid, 3-[(trimethylsilyl)oxy]-, trimethylsilyl ester	219, 177, **147**, 133, 116, 73
18	20.4	heptenoic acid, trimethylsilyl ester	185, 129, 117, **75**
19	20.5	heptanoic acid, trimethylsilyl ester	187, 131, 117, **75**
20	20.7	pentasiloxane, dodecamethyl-	369, 281, 207, 147, **73**
21	21.3	pentasiloxane, dodecamethyl-	369, 353, **281**, 265, 249, 207, 147, 43
22	21.4	unknown	**293**, 277, 205, 189, 146, 130,
23	21.7	benzoic acid, trimethylsilyl ester	194, **179**, 135, 105, 77, 51
24	21.8	octenoic acid, trimethylsilyl ester	199, 129, 117, **75**
25	21.9	octanoic acid, trimethylsilyl ester	201, 145, 129, 117, **75**
26	22.3	1-tridecene	182, 125, 111, 97, 83, 69, **55**
27	22.7	butanedioic acid, bis(trimethylsilyl) ester	247, 172, **147**, 129, 73
28	23.2	nonenoic acid, trimethylsilyl ester	**215**, 145, 129, 117, 73
29	23.3	nonanoic acid, trimethylsilyl ester	230, **215**, 145, 129, 117, 73
30	23.4	hexasiloxane, tetradecamethyl-	443, 355, 281, 221, 147, **73**
31	23.8	tetradecane	198, 127, 113, 99, 85, 71, **57**
32	23.9	pentanedioic acid, bis(trimethylsilyl) ester	261, 233, 204, 158, **147**, 129, 116, 97, 73
33	24.4	decenoic acid, trimethylsilyl ester	227, 145, 129, 117, **75**
34	24.5	decanoic acid, trimethylsilyl ester	**229**, 145, 129, 117, 73
35	25.2	hexanedioic acid, bis(trimethylsilyl) ester	275, 217, 204, 172, 159, 147, 129, 111, **73**
36	25.6	undecenoic acid, trimethylsilyl ester	241, 145, 129, 117, 96, **75**
37	25.7	undecanoic acid, trimethylsilyl ester	**243**, 145, 129, 117, 95, 73
38	26.2	1-hexadecene	224, 125, 111, 97, 83, 69, **55**
39	26.3	hexadecane	226, 127, 113, 99, 85, 71, **57**
40	26.4	heptanedioic acid, bis(trimethylsilyl) ester	289, 217, 204, 186, 173, 155, 147, 125, 97, **73**
41	26.8	dodecenoic acid, trimethylsilyl ester	255, 201, 145, 129, 117, **73**
42	26.9	dodecanoic acid, trimethylsilyl ester	**257**, 201, 145, 129, 117, 73
43	27.0	cyclooctasiloxane, hexadecamethyl-	415, 401, 355, 281, 221, 147, **73**
44	27.3	1-heptadecene	238, 125, 111, 97, 83, 69, **55**
45	27.4	octanedioic acid, bis(trimethylsilyl) ester	303, 217, 187, 169, 147, 129, 117, **73**
46	27.8	tridecenoic acid, trimethylsilyl ester	269, 145, 129, 117, **73**
47	27.9	tridecanoic acid, trimethylsilyl ester	271, 145, 129, 117, **73**
48	28.5	nonanedioic acid, bis(trimethylsilyl) ester	317, 217, 201, 147, 129, 117, 97, **73**
49	28.9	tetradecenoic acid, trimethylsilyl ester	283, 145, 129, 117, **73**
50	29.0	tetradecanoic acid, trimethylsilyl ester	300, **285**, 145, 132, 117, 73
51	29.4	1-nonadecene	266, 139, 125, 111, 97, 83, 69, **55**
52	29.5	decanedioic acid, bis(trimethylsilyl) ester	331, 315, 215, 204, 185, 166, 147, 129, 117, **73**
53	29.9	pentadecenoic acid, trimethylsilyl ester	297, 145, 129, 117, **73**

54	30.0	pentadecanoic acid, trimethylsilyl ester	299, 145, 129, 117,**73**
55	30.5	eicosane	282, 127, 113, 99, 85, 71, **57**
56	30.7	hexadecenoic acid, trimethylsilyl ester	311, 145, 129, 117, **73**
57	30.9	hexadecanoic acid, trimethylsilyl ester	**313**, 145, 129, 117, 73
58	31.3	dodecanedioic acid, bis(trimethylsilyl) ester	359, 243, 217, 204, 129, 117, **73**
59	31.7	heptadecenoic acid, trimethylsilyl ester	325, 145, 129, 117, **73**
60	31.8	heptadecanoic acid, trimethylsilyl ester	342, **327**, 201, 145, 132, 117, 73
61	32.3	docosane	310, 127, 113, 99, 85, 71, **57**
62	32.4	octadecenoic acid, trimethylsilyl ester	339, 145, 129, **117**, 97, 75
63	32.7	octadecanoic acid, trimethylsilyl ester	341, 145, 129, 117, **73**
64	33.0	tetradecandioic acid, trimethylsilyl ester	387, 271, 217, 204, 147, 129, 117, **73**
65	33.5	nonadecanoic acid, trimethylsilyl ester	355, 145, 132, **117**, 97, 73
66	33.9	tetracosane	338, 127, 113, 99, 85, 71, **57**
67	34.2	eicosanoic acid, trimethylsilyl ester	384, **369**, 341, 201, 145, 132, 117, 73
68	35.4	hexacosane	127, 113, 99, 85, 71, **57**
69	36.9	octacosane	141, 127, 113, 99, 85, 71, **57**
70	38.5	nonacosane	131, 127, 113, 99, 85, 71, **57**

Table S.14 List of the main pyrolysis products in the chromatograms obtained in the Py-GC-MS analysis of the extraction residues of both PP-0w and PP-4w (**Figure 17**).

#	t_r	Peak identification	Main ions (*m*/*z*)
1	2.3	1-propene	41
2	2.6	n-pentane	72, 57, **43**
3	3.0	2-methyl-pentane	84, 71, 57, **43**
4	3.2	2-methyl-1-pentene	84,69, **56**, 41
5	3.8	2,4-dimethyl-1-pentene	98,83, **56**, 41
6	4.4	2-methyl-1,5-hexadiene	95, **81**, 67, 55, 39
7	5.0	3-methyl-2,4-hexadiene	96, **81**, 67, 53, 39
8	6.6	4-methyl-2-heptene	112, 97, 83, **69**, 55, 41
9	6.8	4-methylheptane	114, **70**, 55, 43
10	7.0	2,5-dimethyl-1,5-hexadiene	110, **95**, 67, 55, 39
11	8.1	2,4-dimethylheptane	85, 71, 57, **43**
12	8.3	1,3,5-trimethylcyclohexane	126, **111**, 69, 55, 41
13	8.6	2,4-dimethyl-1-heptene	126, 83, 70, 55, **43**
14	8.8	1,3,5-trimethylcyclohexane (isomer)	126, **111**, 69, 55, 41
15	9.0	2,4-dimethyl-1,6-heptadiene	124, **109**, 81, 67, 55, 41
16	9.4	2,4,6-trimethyl-1-heptene	140, 83, 69, 55, **43**
17	9.7	2,4,6-trimethyl-1,6-heptadiene	123, **82**, 67, 55, 41
18	10.7	4,6-dimethyl-2-nonene	154, 111, 85, **69**, 55, 41
19	11.5	2,4,6-trimethyl-1-nonene (meso form)	125, 111, 83, **69**, 57, 43
20	11.6	2,4,6-trimethyl-1-nonene (racemic form)	125, 111, 83, **69**, 57, 43
21	12.0	2,4,6,8-tetramethyl-1-nonene (racemic form)	182, 125, 111, 83, **69**, 57, 43
22	12.2	2,4,6,8-tetramethyl-1,8-nonadiene (racemic form)	180, 165, 123, 109, 96, 83, **69**, 55, 41
23	13.4	2,4,6,8-tetramethyl-1-undecene (isotactic)	154, 111, 85, **69**, 55, 43
24	13.5	2,4,6,8-tetramethyl-1-undecene (syndiotactic)	154, 111, 85, **69**, 55, 43
25	13.7	2,4,6,8,10-pentamethyl-1-undecene (syndiotactic)	224, 168, 125, 111, 97, 83, **69**, 57, 43
26	13.9	2,4,6,8,10-pentamethyl-1,10-undecadiene (syndiotactic)	123, 109, 95, 83, **69**, 55, 41
27	14.2	unknown	153, 111, 97, 85, **69**, 57, 41

28	14.7	2,4,6,8,10-pentamethyl-1,14-tridecadiene	250, 123, 109, 95, 83, **69**, 55, 41
29	14.8	2,4,6,8,10-pentamethyl-1-tridecene (isotactic)	252, 153, 125, 111, 97, 83, **69**, 57, 43
30	15.0	unknown	196, 153, 125, 111, 97, 83, **69**, 57, 43
31	15.1	unknown	153, 125, 111, 97, 83, **69**, 57, 43
32	15.2	2,4,6,8,10,12-hexamethyl-1,12-tridecadiene (syndiotactic)	264, 165, 123, 109, 95, 83, **69**, 55, 41
33	15.9	2,4,6,8,10,12-hexamethyl-1,14-pentadecadiene	292, 151, 123, 109, 97, 83, **69**, 55, 41
34	16.1	2,4,6,8,10,12-hexamethyl-1-pentadecene	294, 153, 125, 111, 97, 83, **69**, 57, 43
35	16.2	unknown	294, 153, 125, 111, 97, 83, **69**, 57, 43
36	16.5	2,4,6,8,10,12,14-heptamethyl-1,14-pentadecadiene	306, 165, 123, 109, 97, 83, **69**, 55, 41
37	17.1	2,4,6,8,10,12,14-heptmethyl-1,16-heptadecadiene	334, 151, 123, 109, 97, 83, **69**, 55, 41
38	17.2	2,4,6,8,10,12,14-heptamethyl-1-heptadecene	336, 153, 125, 111, 97, 83, **69**, 57, 43
39	17.5	2,4,6,8,10,12,14,16-octamethyl-1,16-eptadecadiene	348, 165, 123, 109, 97, 83, **69**, 55, 41
40	18.1	2,4,6,8,10,12,14,16-octamethyl-1,18-nonadecadiene	376, 151, 125, 109, 97, 83, **69**, 55, 41
41	18.2	2,4,6,8,10,12,14,16-octamethyl-1-nonadecene	378, 153, 125, 111, 97, 83, **69**, 57, 43
42	18.5	2,4,6,8,10,12,14,16,18-nonamethyl-1,18-nonadecadiene	390, 165, 125, 109, 97, 83, **69**, 55, 41
43	19.0	2,4,6,8,10,12,14,16,18-nonamethyl-1,20-henicosadiene	418, 153, 125, 111, 97, 83, **69**, 55, 41
44	19.1	2,4,6,8,10,12,14,16,18-nonamethyl-1-henicosene	153, 139, 125, 111, 97, 83, **69**, 57, 43
45	19.4	2,4,6,8,10,12,14,16,18,20-decamethyl-1,20-henicosadiene	432, 165, 125, 109, 97, 83, **69**, 55, 43
46	19.8	2,4,6,8,10,12,14,16,18,20-decamethyl-1,22-tricosadiene	460, 153, 125, 111, 97,83, **69**, 55, 41
47	19.9	2,4,6,8,10,12,14,16,18,20-decamethyl-1-tricosene	153, 139, 125, 111, 97, 83, **69**, 57, 43
48	20.3	2,4,6,8,10,12,14,16,18,20,22-undecamethyl-1,22-tricosadiene	474, 125, 111, 97, 83, **69**, 55, 43
49	20.8	2,4,6,8,10,12,14,16,18,20,22-undecamethyl-1,24-pentacosadiene	502, 153, 125, 111, 97, 83, **69**, 55, 43
50	20.9	2,4,6,8,10,12,14,16,18,20,22-undecamethyl-1-pentacosene	153, 139, 125, 111, 97, 83, **69**, 57, 43
51	21.3	2,4,6,8,10,12,14,16,18,20,22,24-dodecamethyl-1,24-pentacosadiene	516, 153, 125, 111, 97, 83, **69**, 55, 43
52	21.9	2,4,6,8,10,12,14,16,18,20,22,24-dodecamethyl-1,26-heptacosadiene	544, 153, 125, 111, 97, 83, **69**, 55, 43
53	22.1	2,4,6,8,10,12,14,16,18,20,22,24-dodecamethyl-1-heptacosene	153, 139, 125, 111, 97, 83, **69**, 57, 43
54	22.6	2,4,6,8,10,12,14,16,18,20,22,24,26-tridecamethyl-1,26-heptacosadiene	560, 165, 125, 111, 97, 83, **69**, 57, 43
55	23.5	2,4,6,8,10,12,14,16,18,20,22,24,26-tridecamethyl-1,28-nonacosadiene	588, 153, 125, 111, 97, 83, **69**, 57, 43
56	23.7	2,4,6,8,10,12,14,16,18,20,22,24,26-tridecamethyl-1-nonacosene	153, 139, 125, 111, 97, 83, **69**, 57, 43
57	24.5	2,4,6,8,10,12,14,16,18,20,22,24,26,28-tetradecamethyl-1,28-nonacosadiene	153, 139, 125, 111, 97, 83, **69**, 57, 43

Table S.15 List of the main pyrolysis products in the chromatograms obtained in the Py-GC-MS analysis of the extraction residues of both PS-0w and PS-4w (**Figure 18**).

#	t_r	Peak identification	Main ions (*m/z*)
1	6.82	toluene	92, **91**, 65
2	9.44	styrene	**104**, 78, 63, 51
3	10.15	allylbenzene	**117**, 103, 91, 65
4	10.59	α-methylstyrene	**118**, 103, 91, 78, 63, 51
5	11.27	3-butenylbenzene	132, 104, **91**, 65
6	11.41	(1-methylenepropyl)-benzene	132, **117**, 103, 91, 77, 63, 51
7	14.95	1,2-diphenylethane	182, **91**, 65
8	15.14	propane-1,2-diyldibenzene	196, **105**, 91, 77, 65
9	15.71	1,1'-(1,3-propanediyl)bis-benzene	196, 105, **92**, 77, 65, 51
10	16.07	Stilbene	180, **179**, 165, 152, 102, 89, 76
11	16.11	3-butene-1,3-diyldibenzene (styrene dimer)	208, 193, 130, 115, 104, **91**, 77, 65
12	16.16	1-pentene-2,4-diyldibenzen	222, 194, 179, 115, **105**, 91, 77
13	16.64	(E)-1-butene-1,4-diyldibenzene	208, **117**, 115, 91, 65
14	17.01	hexa-1,5-diene-2,5-diyldibenzene	234, 143, **130**, 115, 104, 91, 77, 65
15	19.51	5-hexene-1,3,5-triyltribenzene (styrene trimer)	312, 207, 194, 117, **91**, 77

Table S.16 List of the main pyrolysis products in the chromatograms obtained in the Py-GC-MS analysis of the extraction residues of both PET-0w and PET-4w (**Figure S.16**).

#	t_r	Peak identification	Main ions (*m/z*)
1	2.27	carbon dioxide	**44**
2	4.13	benzene	**78**, 63, 51
3	8.88	ethylbenzene	106, **91**, 77, 65, 51
4	9.37	styrene	**104**, 78, 63, 51
5	10.35	benzaldehyde	106, **105**, 77, 51
6	10.66	phenol	**94**, 79, 66, 55, 39
7	11.27	benzeneacetaldehyde	120, **91**, 65, 51
8	11.51	acetophenone	120, **105**, 77, 51
9	12.15	vinyl benzoate	148, **105**, 77, 51
10	12.87	benzoic acid	122, **105**, 77, 51
11	13.00	benzoic acid trimethylsilyl ester	194, **179**, 135, 105, 77
12	14.03	biphenyl	**154**, 131, 115, 76
13	14.82	unknown	**147**, 119, 104, 91, 76
14	15.15	divinyl terephthalate	**175**, 147, 132, 104, 76
15	15.54	4-(vinyloxycarbonyl) benzoic acid	**149**, 121, 76, 65
16	16.05	stilbene	**180**, 179, 165, 152, 102, 89, 76
17	16.31	9H-fluoren-9-one	**180**, 152, 126, 76
18	16.99	unknown	198, **181**, 152, 76
19	18.24	ethan-1,2-diyldibenzoate	227, **105**, 77, 51
20	18.47	unknown	**230**, 198, 181, 152, 115

21	20.12	2-(benzoyloxy) ethyl vinyl terephthalate	**297**, 149, 105, 77
22	22.52	ethan-1,2-diyl divinyl diterephthalate	**367**, 325, 296, 175, 162, 104

Table S.17 List of the main pyrolysis products in the chromatograms obtained in the Py-GC-MS analysis of the extraction residues of both 0w and 4w LDPE and HDPE (**Figure 19**).

#	t_r	Peak identification	Main ions (*m/z*)
1	3.16	1-hexene	84, 69, **56**, 41
2	4.60	1-heptene	98, 83, 70, 56, **41**
3	4.79	heptane	100, 71, 57, **43**
4	7.23	1,7-octadiene	110, 95, 82, **67**, 54, 41
5	7.43	1-octene	112, 97, 83, 70, **55**, 41
6	7.62	octane	114, 85, 71, 57, **43**
7	9.21	1,8-nonadiene	109, 96, 81, 67, **55**, 41
8	9.34	1-nonene	126, 97, 83, 69, **56**, 41
9	9.47	nonane	128, 99, 85, 71, 57, **43**
10	10.57	1,9-decadiene	110, 95, 81, 67, **55**, 41
11	10.66	1-decene	140, 111, 97, 83, 70, 55, **41**
12	10.75	decane	142, 113, 99, 85, 71, **57**, 43
13	11.59	1,10-undecadiene	124, 109, 95, 81, 67, 55, **41**
14	11.67	1-undecene	154, 125, 111, 97, 83, 70, 55, **41**
15	11.74	undecane	156, 98, 85, 71, **57**, 43
16	12.45	1,11-dodecadiene	138, 124, 109, 95, 81, 67, **55**, 41
17	12.51	1-dodecene	168, 140, 125, 111, 97, 83, 69, 55, **41**
18	12.57	dodecane	170, 128, 85, 71, **57**, 43
19	13.20	1,12-tridecadiene	123, 109, 95, 81, 67, **55**, 41
20	13.26	1-tridecene	125, 111, 97, 83, 69, 55, **41**
21	13.31	tridecane	184, 99, 85, 71, **57**, 43
22	13.90	1,13-tetradecadiene	123, 109, 96, 81, 67, **55**, 41
23	13.95	1-tetradecene	125, 111, 97, 83, 69, 55, **41**
24	14.00	tetradecane	198, 99, 85, 71, **57**, 43
25	14.54	1,14-pentadecadiene	123, 109, 96, 81, 67, **55**, 41
26	14.59	1-pentadecene	210, 125, 111, 97, 83, 69, 55, **41**
27	14.63	pentadecane	212, 113, 99, 85, 71, **57**, 43
28	15.15	1,15-hexadecadiene	123, 109, 96, 82, 67, **55**, 41
29	15.19	1-hexadecene	224, 125, 111, 97, 83, 69, **55**, 41
30	15.22	hexadecane	226, 99, 85, 71, **57**, 43
31	15.73	1,16-heptadecadiene	137, 123, 109, 96, 82, 69, **55**, 41
32	15.76	1-heptadecene	238, 139, 125, 111, 97, 83, 69, **55**, 41
33	15.80	heptadecane	240, 99, 85, 71, **57**, 43
34	16.27	1,17-octadecadiene	123, 109, 96, 82, 69, **55**, 41
35	16.30	1-octadecene	252, 125, 111, 97, 83, 69, **55**, 41
36	16.34	octadecane	254, 99, 85, 71, **57**, 43
37	16.78	1,18-nonadecadiene	137, 123, 109, 96, 82, 69, **55**, 41
38	16.82	1-nonadecene	139, 125, 111, 97, 83, 69, 55, **43**
39	16.85	nonadecane	268, 127, 113, 99, 85, 71, **57**, 43
40	17.29	1,19-eicosadiene	137, 123, 109, 96, 82, 69, **55**, 41
41	17.31	1-eicosene	139, 125, 111, **97**, 83, 69, 57, 43
42	17.34	eicosane	127, 113, 99, 85, 71, **57**, 43
43	17.75	1,20-heneicosadiene	137, 123, 109, 96, 82, 69, **55**, 41
44	17.78	1-heneicosene	139, 125, 111, **97**, 83, 69, 55, 43

45	17.81	heneicosane	113, 97, 85, 71, **57**, 43
46	18.21	1,21-docosadiene	151, 137, 123, 109, 96, 82, 69, **55**, 41
47	18.23	1-docosene	139, 125, 111, 97, 83, 69, **57**, 43
48	18.25	docosane	310, 99, 85, 71, **57**, 43
49	18.64	1,22-tricosadiene	137, 123, 109, 96, 82, 69, **55**, 41
50	18.66	1-tricosene	322, 125, 111, 97, 83, 69, **55**, 43
51	18.68	tricosane	324, 113, 97, 85, 71, **57**, 43
52	19.06	1,23-tetracosadiene	334, 137, 123, 109, 96, 83, 69, **55**, 41
53	19.08	1-tetracosene	336, 139, 125, 111, 97, 83, 69, **57**, 43
54	19.47	1-pentacosene	350, 139, 125, 111, 97, 83, 69, **57**, 43
55	19.87	1-hexacosene	364, 139, 125, 111, 97, 83, 69, **57**, 43
56	20.28	1-heptacosene	378, 139, 125, 111, 97, 83, 71, **57**, 43
57	21.22	1-octacosene	139, 125, 111, 97, 83, 71, **57**, 43
58	21.78	1-nonacosene	139, 125, 111, 97, 83, 71, **57**, 43
59	22.43	1-triacontene	139, 125, 111, 97, 83, 71, **57**, 43
60	23.18	1-hentriacontene	139, 125, 111, 97, 83, 71, **57**, 43
61	24.07	1-dotriacontane	139, 125, 111, 97, 83, 69, **57**, 43
62	24.48	unknown	153, 125, 111, 97, 83, **69**, 57, 43
63	25.13	1-tritriacontene	139, 125, 111, 97, 83, 71, **57**, 43
